# Construction of a highly error-prone DNA polymerase for developing organelle mutation systems

**DOI:** 10.1093/nar/gkaa929

**Published:** 2020-11-02

**Authors:** Junwei Ji, Anil Day

**Affiliations:** School of Biological Sciences, Faculty of Biology, Medicine and Health, The University of Manchester, Manchester M13 9PT, UK; School of Biological Sciences, Faculty of Biology, Medicine and Health, The University of Manchester, Manchester M13 9PT, UK

## Abstract

A novel family of DNA polymerases replicates organelle genomes in a wide distribution of taxa encompassing plants and protozoans. Making error-prone mutator versions of gamma DNA polymerases revolutionised our understanding of animal mitochondrial genomes but similar advances have not been made for the organelle DNA polymerases present in plant mitochondria and chloroplasts. We tested the fidelities of error prone tobacco organelle DNA polymerases using a novel positive selection method involving replication of the phage lambda *cI* repressor gene. Unlike gamma DNA polymerases, ablation of 3′–5′ exonuclease function resulted in a modest 5–8-fold error rate increase. Combining exonuclease deficiency with a polymerisation domain substitution raised the organelle DNA polymerase error rate by 140-fold relative to the wild type enzyme. This high error rate compares favourably with error-rates of mutator versions of animal gamma DNA polymerases. The error prone organelle DNA polymerase introduced mutations at multiple locations ranging from two to seven sites in half of the mutant *cI* genes studied. Single base substitutions predominated including frequent A:A (template: dNMP) mispairings. High error rate and semi-dominance to the wild type enzyme *in vitro* make the error prone organelle DNA polymerase suitable for elevating mutation rates in chloroplasts and mitochondria.

## INTRODUCTION

Eukaryotic cells contain essential multi-copy organelle genomes in chloroplasts and mitochondria. Stable maintenance of these extra-nuclear genomes is required for the proper functioning of mitochondria and chloroplasts. Mutants arising from mutations in organelle genomes have provided a valuable resource to study the roles of organelle genes (1,2). In animals and fungi, error-prone versions of gamma DNA polymerase have been used to elevate mutation rates in mitochondria to advance our understanding of mitochondrial genomes (3–5). Use of error-prone mutator DNA polymerases have led to new discoveries on the replication mechanisms and selective forces acting on animal mitochondrial genomes, and the impact of elevated mutation rates on organism biology including aging (6–10). By comparison, our knowledge of these fundamental processes in the organelles of plants is limited. The evolutionary mutation rates of plant organelle genomes are much lower than those observed in plant nuclear genes (2,11,12). To advance our understanding of plant organelle genomes by elevating the mutation rate with mutator DNA polymerases requires the construction and characterisation of error-prone versions of plant organelle DNA polymerases.

Plant organelles contain a novel family of DNA polymerases, named Plant Organellar DNA Polymerases (POPs) (13). The name POP now covers plant and protist organelle DNA polymerases to reflect the widespread distribution of POPs in a diverse range of algae and protozoans (13–15). POPs and gamma DNA polymerases are distantly related members of the DNA polymerase A family (14). In common with other DNA polymerases, POPs contain 5′-3′ DNA polymerisation and 3′–5′ exonuclease (proof-reading) domains in a single polypeptide (13,16,17). POPs are considered to be the sole enzymes responsible for replication of the mitochondrial and chloroplast genomes in plants. They are highly processive enzymes (17–19) with a novel combination of activities including strand-displacement (18,19), translesion synthesis (19), microhomology-mediated-end-joining (20) and 5′ deoxyribose phosphate removal (18,21).

**Figure 1.**
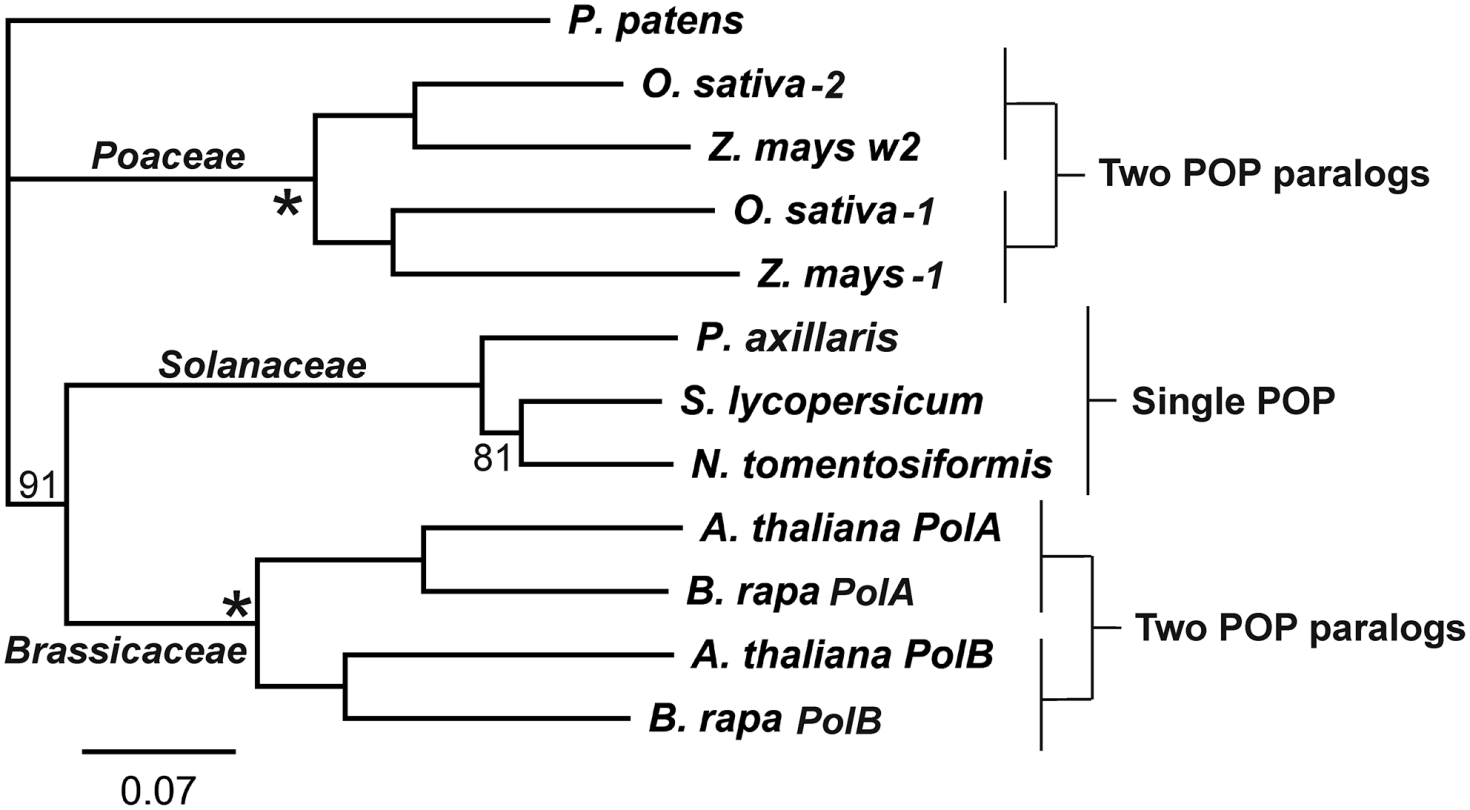
Neighbour-joining consensus tree of indicated POP sequences. Bootstrap values (1000 replicates) were 100% unless indicated otherwise. Sequences were retrieved from GenBank: *Arabidopsis thaliana* (PolA AEE32595; PolB AEE76393), *Brassica rapa* (PolA XP_009144938; PolB XP_009145617), *Nicotiana tomentosiformis* (XP_009610361), *Oryza sativa* (1. BAT04060; 2. XP_015636766), *Physcomitrella patens* (PNR49997), *Solanum lycopersicum* (XP_004244135), *Zea mays* (1. XP_020401293; w2 AQK46502). *Petunia axillaris* (Peaxi162Scf00450g00842.1) was from the SOL Genomics Network. *P. patens* was used as the outgroup. Indicated are taxa containing a single POP or two divergent POP paralogs. Asterisks (*) indicate duplication events responsible for POP paralogs. Scale bar: amino acid substitutions per site.

Plant POPs are expressed from nuclear genes and targeted to organelles (16,17,22). Our phylogenetic analysis of POPs (Figure 1, Supplementary Figure S1) revealed two patterns of POP distribution in angiosperms. Dicot families such as the Solanaceae contain a single POP gene in diploid (2*n*) species such as *Solanum lycopersicum*, *Nicotiana tomentosiformis* and *Petunia hybrida*. The second group of plants contain two divergent POP genes whose products share 70–76% amino acid identity in taxonomically distant dicot and monocot families exemplified by the *Brassicaceae* and *Poaceae* families (Figure 1, Supplementary Figure S1). The gene duplications giving rise to these POP paralogs in the *Brassicaceae* and *Poaceae* took place after their divergence from a common ancestor. Plant POPs from *Nicotiana tabacum* (*Solanaceae*) and *Arabidopsis thaliana* (*Brassicaceae*) were shown to be dual targeted to both organelles (17,22,23). Single gene knockouts of POP genes are viable in *A. thaliana* (24) but not in *Zea mays* (*Poaceae*), where chloroplast DNA but not mitochondrial DNA was reduced to low lethal amounts (25,26). This difference indicates redundancy of POP genes in *A. thaliana* but not in *Zea mays*. Despite this redundancy, differences have been found between the two *A. thaliana* POPs (AtPolA and AtPolB), with respect to their enzymatic properties (27), roles in DNA replication versus repair (24,27,28), interactions with other proteins (29) and relative importance in mitochondria versus chloroplasts (30).

To develop an error-prone mutator POP we chose to engineer an enzyme from the *Solanaceae*. Use of a *Solanaceous* POP has the advantage of engineering the sole enzyme responsible for the DNA polymerase-related replication/repair activities in plant organelles. We chose a POP from *Nicotiana tabacum* (tobacco), which is the leading model for transgenic research on organelle genomes (31,32). *N. tabacum* is allotetraploid (4*n*) resulting from a relatively recent fusion between diploid (2*n*) *N. tomentosiformis* and *N. sylvestris* parents (33,34). To evaluate the impact of amino acid substitutions on the replication fidelities of DNA polymerases we developed a novel mutation screening assay based on the positive selection scheme described by Nilsson *et al.* (1983) (35). In the assay a single stranded stretch of the bacteriophage lambda *cI* gene encoding the CI repressor protein is replicated by a DNA polymerase *in vitro* before transformation of the plasmid into *E. coli*. Replication errors resulting in loss of function prevent CI repressor binding to its target sequence upstream of the tetracycline resistance gene. This approach gives rise to tetracycline-resistant colonies containing plasmids with mutations in the *cI* gene that can be sequenced and compared to the large data set of previously mapped loss-of-function mutations in the *cI* gene (36–39). At high plating densities, positive selection has the advantage of ease of identifying resistant mutant colonies compared to colony screening methods based on colour (40–42). Here we used the assay to construct and characterise a highly error prone *N. tabacum* POP suitable for elevating mutation rates in organelles.

## MATERIALS AND METHODS

### Sequence analysis software

*In silico* vector assembly and sequence analyses were carried out using SnapGene (San Diego), Vector NTI Advance (Thermo Fisher Scientific, Paisley) and Geneious Prime (Biomatters, Auckland). Protein alignments from Geneious Aligner were used in Geneious Tree Builder to assemble neighbour-joining trees (43).

### Cloning, overexpression and purification of recombinant NtPOP^tom^ enzymes

General methods for recombinant DNA work and molecular biology procedures including media composition and buffers were from Sambrook *et al.* (1989) (44). The NtPOP^tom^ WT cDNA was isolated from *N. tabacum* var Petit Havana. The modified cDNA sequence is shown in Supplementary Figure S2. The amino acid substitutions in the exonuclease and polymerisation domains were introduced into the coding region using the Q5 site directed mutagenesis kit (New England Biolabs). The polymerisation domain was excised by replacing the internal Nde I and Pst I fragment in the NtPOP^tom^ cDNA with annealed oligos delNdeIPstI-F and delNdeIPstI-R (Supplementary Table S4). Coding sequences were cloned into pET30b (Invitrogen) and expressed in Novagen Rosetta 2(DE3) cells (Sigma-Aldrich, Southampton, UK). Recombinant protein expression was induced with 1 mM IPTG for 3 h in cells grown in Terrific Broth (Sigma-Aldrich) containing 50 μg/ml kanamycin and 37 μg/ml chloramphenicol. All next steps were done on ice. Sedimented cells were resuspended in chilled buffer P (50 mM bis–tris pH 8.0, 150 mM NaCl and 1 mM EDTA) supplemented with 0.1% Triton X100 (w/v), 1 mg/ml lysozyme, protease inhibitor cocktail (Roche UK, Welwyn Garden City, UK) and lysed by sonication. RNase A (10 μg/ml) and DNase I (5 μg/ml) were added to the lysate and incubated for 15 min. The mixture was spun at 21 000 × g for 15 min. The protein was purified using a Strep-Tactin^®^-XT purification column (IBA Life Sciences, Goettingen, Germany) and stored in buffer P containing 50% (v/v) glycerol and 1 mM dithiothreitol at −20°C. The five N-terminal amino acids of the purified 99 kDa NtPOP^tom^ WT enzyme were determined by Edman degradation (AltaBioscience, Redditch, UK).

### DNA replication assays

We followed the protocol of Tveit and Kristensen (2001) substituting PicoGreen (45) with Quantifluor One dsDNA fluorescence dye (Promega, Southampton). Synthesis of double-stranded DNA was from a 35 base oligonucleotide (M13-F, Supplementary Table S4) annealed to single-stranded M13mp18 DNA in buffer R (10 mM Tris–HCl pH 8.0, 100 mM NaCl, 2.5 mM MgCl_2_, 1 mM DTT, 333 μM dNTPs and 100 μg/ml bovine serum albumin). Reactions at 30°C were initiated by the addition of enzyme and terminated by adding EDTA to 8 mM and placing on ice. Each reaction in 30 μl contained 12–400 fmol of purified recombinant DNA polymerase with the primed M13mp18 template in excess apart from competition experiments using 600 fmol of WT enzyme when the template was saturated. Double stranded DNA was quantified using the Quantifluor One dsDNA fluorescence dye and a Synergy HI Multi-Mode Microplate Reader (BioTek Instruments) set at 504 nm_Ex_/531 nm_Em_.

Gapped DNA was prepared using the competing oligonucleotide-method (42). pUN121 (35) was nicked with Nb.Bpu10I (ThermoFisher Scientific, Paisley) and mixed with three competing oligonucleotides (Supplementary Table S4) complementary to the nicked 162 base non-coding strand in 50-fold molar excess. The mixture in nicking buffer (10 mM Tris–HCl pH 8.5, 10 mM MgCl_2_, 100 mM KCl and BSA 100 μg/ml) was heated to 95°C and cooled gradually to 75°C over 30 min and then left to cool to room temperature. Competitor oligonucleotides were removed using QIAquick purification columns (QIAGEN, Manchester). Gapped plasmids were purified using benzoylated naphthoylated DEAE cellulose (Sigma-Aldrich, Poole) as described by Wang and Hays (2001) (46). Purified gapped plasmid was digested with Hind III before use in replication assays to linearize any double-stranded DNA contaminating the gapped plasmids. This step effectively removes contaminating double-stranded DNA from the bacterial colony screen because linear DNA is an ineffective transformation substrate in *E. coli*. The gapped plasmid was ready for use after removal of Hind III using a QIAquick purification column. Replication of gapped plasmid was for 15 min in 30 μl of buffer R at 30°C for recombinant POP enzymes and 72°C for Taq DNA Pol. Replication was verified using Hind III digestion. Hind III linearises the replicated plasmids but not the gapped plasmid (Supplementary Figure S3). The replicated plasmids were transformed into DH5α competent cells (New England Biolabs). Transformed cells were plated on LB agar medium containing either 100 μg/ml ampicillin or 15 μg/ml tetracycline and incubated at 37°C to visualise colonies.

### Mutant frequency and error rate

Mutant frequency was calculated by dividing the number of tetracycline-resistant colonies by the number of ampicillin resistant colonies after accounting for the difference in plating efficiency. Using a pUN121 plasmid with a loss-of-function mutation in the *cI* gene, the number of colonies on tetracycline medium were 61% of the number obtained on ampicillin medium. The error rate (ER) was calculated by scoring mutations in the coding region containing the well-studied alpha 1 and 5 helices (37,39) in the *cI* gene. ER was determined from the equation ER = MF/(D x P) (41,47) where MF is the mutation frequency of tetracycline resistant colonies resulting from mutations in the alpha 1 and 5 coding regions, D the number of detectable sites in this sequence stretch and P the probability that a mutation in the newly synthesized strand will be expressed. P was determined experimentally. A 5′ phosphorylated oligonucleotide (pUN121_mut) with a 2-base deletion in the Hind III site was annealed and ligated to gapped pUN121. This heteroduplex region was then extended with Taq DNA polymerase in buffer R. A temperature of 30°C was used to prevent strand displacement activity. The replicated plasmid was purified using a QIAquick purification column and treated with Hind III to linearize any pUN121 lacking the heteroduplex at the Hind III site. Following transformation of *E. coli* the ratio of tetracycline to ampicillin colonies provided an estimate of the probability of expression, which was 2.5%. Estimation of detectable sites required identification of base changes at every position in the alpha 1 and 5 coding region that inactivate the CI repressor (Supplementary Figure S4) using published data (37,39). These include 51.3 base substitutions and 99 indels providing a total of 150.3 detectable sites in coding sequences for alpha helices 1 and 5.

### DNA sequencing

Plasmids were purified using the Isolate II kit (Bioline, London) and sequenced (Eurofins Genomics, Wolverhampton) with primers pUN121-F and pUN121-R (Supplementary Table S4). Sequences were analysed using Geneious Prime software (Biomatters, Auckland).

### Protein blot analyses

Bacterial cells were lysed in sample buffer (50 mM Tris–HCl, pH 6.8, 12.5 mM EDTA, 10% (v/v) glycerol, 2% (w/v) SDS, 2% (v/v) ß-mercaptoethanol, 0.1% (w/v) bromophenol blue) and placed in a boiling water bath for 5 minutes. Following centrifugation for 5 min at 14 000 rpm (Eppendorf 5415c, Stevenage) supernatants were fractionated on 10% (w/v) polyacrylamide gels prepared using TGX FastCast acrylamide solutions (Bio-Rad, Hemel Hempstead) in a mini-Protean 3 electrophoresis tank (BioRad) in running buffer (25 mM Tris, 192 mM glycine, 0.1% w/v SDS). Following electrophoresis gels were viewed with the Molecular Imager Gel Doc XR system (BioRad) after UV activation of tri-halo compounds. Proteins from SDS-PAGE gels were transferred using Turbo-Blot Turbo Mini 0.2 μm nitrocellulose transfer packs and the Trans-blot Turbo transfer system (Bio-Rad). Proteins were detected as previously described (48). Primary antibodies used were a monoclonal antibody against Strep-tag II (IBA Lifesciences, Göttingen) and a rabbit polyclonal antibody raised against the peptide NTETGRLSARRPNLQ in the POP polymerisation domain, which was affinity-purified using the same peptide (Eurogentec, Liege). Secondary antibodies linked to alkaline phosphatase (Sigma–Aldrich, Poole, UK) were stained with 5-bromo-4-chloro3-indolyl phosphate/nitro blue tetrazolium (BCIP/NBT) liquid substrate (Sigma-Aldrich, Southampton).

### Statistical analyses

We followed the method of Stone *et al.* (49) involving two tailed chi squared analyses to identify significant differences between base substitution error rates for the POP enzymes.

## RESULTS

### Structure of WT and mutant *N. tabacum* POPs

**Figure 2.**
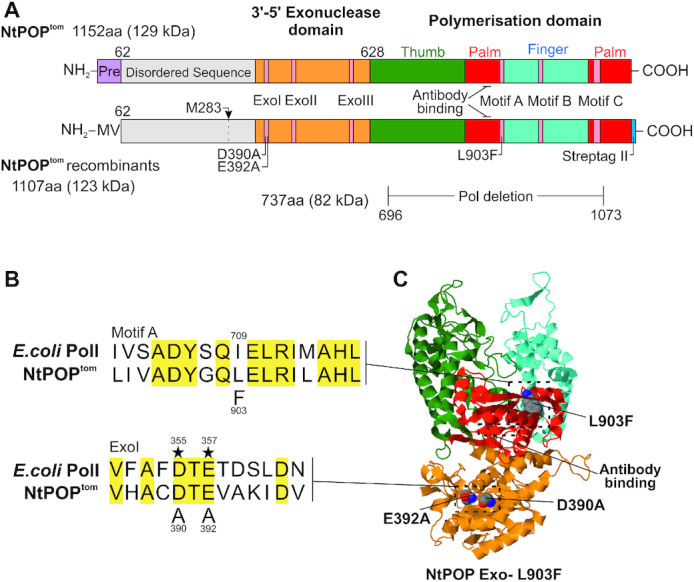
Scheme showing the organisation of NtPOP^tom^ proteins. (**A**) NtPOP^tom^ 1152 amino acid native protein (top) aligned with recombinant proteins (bottom). Shown are N-terminal presequence (Pre), disordered region, exonuclease and polymerisation domains locating D390A, E392 and L903F substitutions, C-terminal Strep-tag II, cleavage site preceding M283 (arrowed) observed in *E. coli* expressed NtPOP^tom^, and region deleted in Pol– enzyme. (**B**) Alignments of motifs A and Exo I in NtPOP^tom^ with *E. coli* DNA Pol I. *E. coli* DNA Pol I residues I709, D355 and E357 align with the substituted L903, D390 and E392 amino acids in NtPOP^tom^. Asterisks (*) indicate amino acids essential for function. (**C**) SWISS MODEL (56) of NtPOP^tom^ based on homology to the Klenow fragment of *E. coli* DNA Pol I (57). Locations of substituted amino acids and antibody binding site are shown.

*N. tomentosiformis* and *N. sylvestris*, the diploid parents of *N. tabacum* (34), contain a single POP enzyme. Whilst *N. tabacum* does not contain POP paralogs, it has inherited the POP orthologs present in its parents. We identify these orthologs as NtPOP^tom^ and NtPOP^sylv^ to indicate their parental origins. NtPOP^tom^ and NtPOP^sylv^ correspond to the NtPol1-like 1 and NtPol1-like 2 proteins in Ono *et al.* (2007) (17), respectively. NtPOP^tom^ (NtPol1-like 1) studied here shares 98% amino acid identity with its parental POP in *N. tomentosiformis*. The domain organisation of the 1152 amino acid NtPOP^tom^ enzyme is shown schematically in Figure 2A. The protein contains a predicted 61 amino acid N-terminal organelle targeting sequence (50) followed by a disordered region of unknown function with low sequence conservation. The disordered regions from NtPOP^tom^ and *A. thaliana* (AtPolB) POPs only share 18% amino acid identity whereas the regions containing the 3′-5′ exonuclease and polymerisation domains share 71% amino acid identity (not shown). The disordered region is not found in other members of the DNA polymerase A family, which includes the first characterised member of the group: *Escherichia coli* DNA Polymerase I (Pol I). NtPOP^tom^ amino acids 352–1152 aligned with amino acids 340 to 925 of the Klenow fragment of *E. coli* DNA Pol I share 24% amino acid identity (Supplementary Figure S5). Within this region are highly conserved sequence motifs located in the 3′–5′ exonuclease (proof-reading) and 5′-3′ polymerisation domains (51). Figure 2A locates exonuclease motifs Exo I-III, and polymerisation domain motifs A–C, on a schematic diagram of the NtPOP^tom^ primary sequence. In Figure 2B, a highly conserved eight amino acid sequence DYSQIELR (52) in motif A of the polymerisation domain in *E. coli* DNA Pol I is aligned with the corresponding region of NtPOP^tom^. Within this DYSQIELR motif in *E. coli* DNA Pol I, substitutions at isoleucine 709 gave rise to an efficient mutator DNA polymerase (53). The equivalent L979F mutation in *Saccharomyces cerevisiae* DNA polymerase zeta also gave rise to a functional and highly error prone enzyme (49). Other amino acids that reduce *E. coli* DNA Pol I replication fidelity include R668, E710 and N845 (54). These residues are conserved in NtPOP^tom^ and represent additional residues that could be targeted to develop an error prone enzyme. Replacement of aspartic acid with alanine in the DYSQIELR motif in a rice POP destroyed DNA synthesis activity (18).

Four recombinant NtPOP^tom^ proteins were expressed in *E. coli*. All lacked the first N-terminal 61 amino acids corresponding to the predicted organelle targeting sequence (50). The changes to the WT protein are summarised in the diagrammatic scheme of the 1107 amino acid recombinant protein in Figure 2A. The protein sequence detailing these changes is provided in Supplementary Figure S6. The N-terminal 61 amino acids were replaced by an initiator methionine followed by a valine for expression in *E. coli*. We refer to the recombinant protein containing the wild type (WT) exonuclease and polymerisation domains as WT. The exonuclease deficient (Exo-) recombinant protein contained D390A and E392A substitutions in the Exo I motif (Figure 2A and B). The corresponding D355A and E357A substitutions in *E. coli* Pol I (Figure 2B) destroy exonuclease activity (55). The Exo-L903F recombinant protein contained a L903F substitution in the polymerisation domain in addition to the D390A and E392A substitutions. The locations of changed amino acids on the 3D-model (56,57) are shown in Figure 2C. Pol- was a defective recombinant enzyme lacking amino acids 696–1073 of the polymerisation domain (Figure 2A). A C-terminal Strep-tag II (58) preceded by a GSGSGS linker facilitated purification. The purified recombinant NtPOP^tom^ enzymes were fractionated by SDS-PAGE on stain-free gels (Bio-Rad) and studied by protein blot analyses using antibodies recognising the POP polymerisation domain and Strep-tag II (supplementary Figure S7).

### DNA synthesis activity of recombinant NtPOP^tom^ enzymes

**Figure 3.**
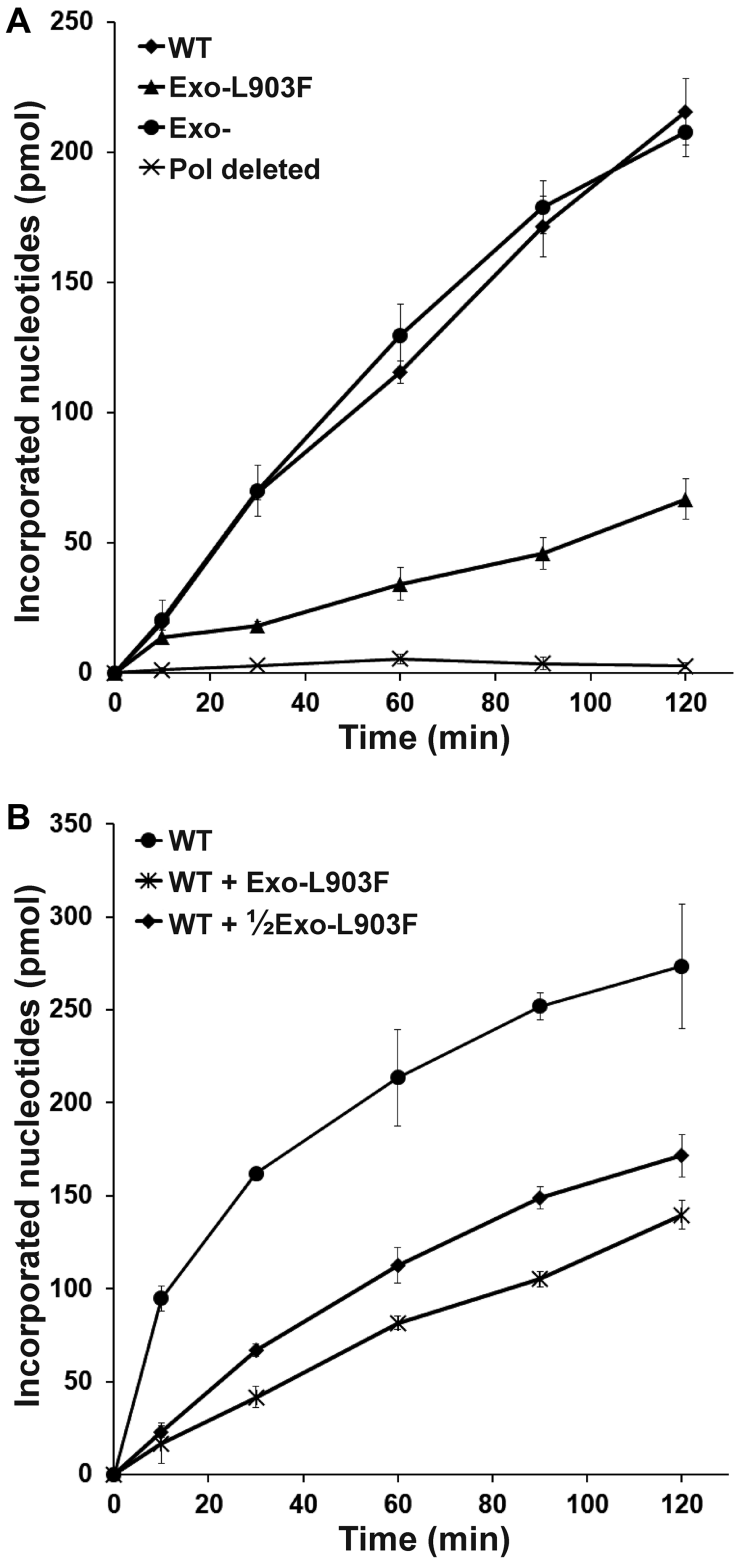
DNA synthesis against time by the recombinant NtPOP^tom^ enzymes. (**A**) Comparison using 400 fmol of the indicated enzymes. (**B**) The influence of adding 300 and 600 fmol of the Exo-L903F enzyme to 600 fmol of the WT enzyme on DNA synthesis rate. Results were from three independent replicates.

DNA synthesis by the four recombinant NtPOP^tom^ enzymes (WT, Exo-, Exo-L903F and Pol-) was measured by replication of M13 single stranded DNA from an annealed 35-mer oligonucleotide. Figure 3A shows the synthesis of double-stranded DNA against time catalysed by the recombinant NtPOP^tom^ enzymes. The replication activities of the WT and Exo- enzymes were indistinguishable. This confirmed that the amino acid substitutions introduced into the exonuclease domain (Figure 2B) did not affect polymerase activity, consistent with the *E. coli* DNA Pol I data (59). DNA synthesis by the Exo-L903F enzyme was reduced by about 70% (Figure 3A, Supplementary Figure S8) reflecting a detrimental effect of the polymerisation domain L903F amino substitution on DNA synthesis. Removal of bacterial DNA polymerases by our purification regime was demonstrated by the negligible rate of DNA synthesis observed using the Pol- protein, which lacks the DNA polymerisation domain responsible for DNA synthesis (Figure 3A).

To assess the potential of the recombinant Exo-L903F enzyme to compete with the WT enzyme during replication of templates a competition experiment was conducted. Different amounts of Exo-L903F were added to a fixed amount of the WT enzyme under conditions where the enzymes were in excess relative to the DNA template. Increasing amounts of Exo-L903F reduced the overall rate of DNA synthesis (Figure 3B) consistent with effective competition between the Exo-L903F and WT enzyme for template replication.

### Genetic screen to estimate DNA polymerase replication fidelity

**Figure 4.**
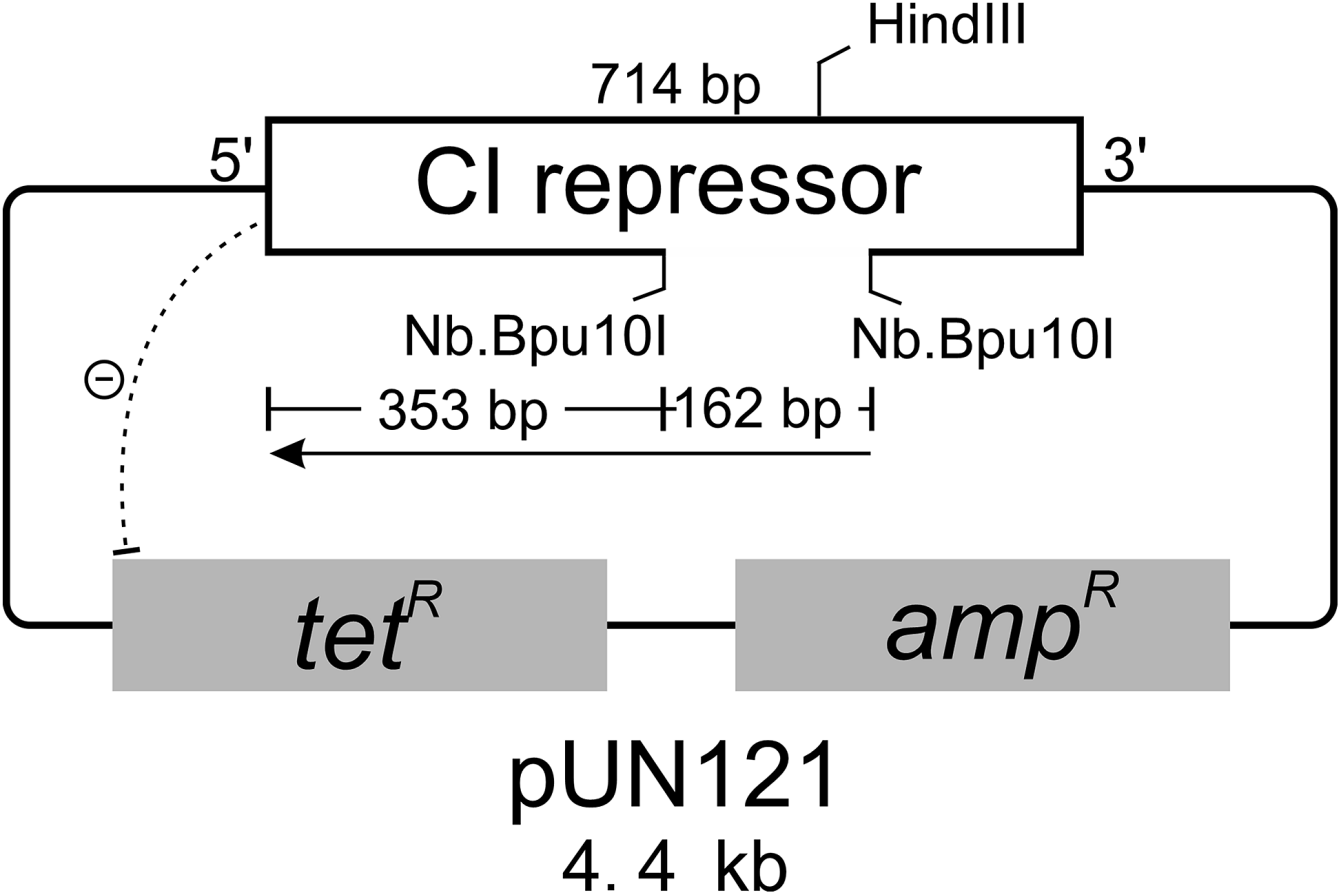
Map of pUN121 (35) showing the coding sequence for the lambda CI repressor, and tet^R^ and amp^R^ genes conferring resistance to tetracycline and ampicillin. The CI repressor prevents expression of tet^R^. A single stranded 162 nucleotide gap (dashed line) on the non-coding strand of the cI gene was made using the nicking enzyme Nb. Bpu10I. The direction of DNA replication is shown and extended 353 nucleotides beyond the gap to the N-terminal coding region of *cI*.

The assay involved replication across the coding sequence of the phage lambda CI repressor in the positive selection vector pUN121 (35), which contains ampicillin (amp^R^) and tetracycline (tet^R^) resistance genes (Figure 4). The CI repressor binds upstream of the tet^R^ gene preventing its expression. Replication errors that inactivate the CI repressor gene in pUN121 allow tet^R^ expression and survival of bacterial colonies on tetracycline medium. The presence of the amp^R^ gene enables the total number of plasmid-containing colonies to be estimated on ampicillin plates. A single-stranded gap in the *cI* gene was prepared by removing 162 nucleotides of the non-coding strand using the nicking enzyme (Nb.Bpu10I) and the competitor oligonucleotide method (42,46). This single-stranded gap is complementary to bases 354 to 515 of the 714 nucleotide *cI* gene and encodes amino acids 119 to 172, which includes the hinge region and residues in the C-terminal domain of the repressor important for dimer formation and cooperative binding of two repressor molecules to two operator sites (38). Replication of the single-stranded gap was towards the N-terminal coding region of the *cI* gene (Figure 4). Continuation of replication beyond the 162 base gap requires strand displacement of the 353 bases to the ATG initiating codon and increases the region of the CI repressor gene replicated to 515 nucleotides. The complementary template strand encodes amino acids 1–118 of the N-terminal DNA binding domain of the CI repressor protein (37,38).

**Figure 5.**
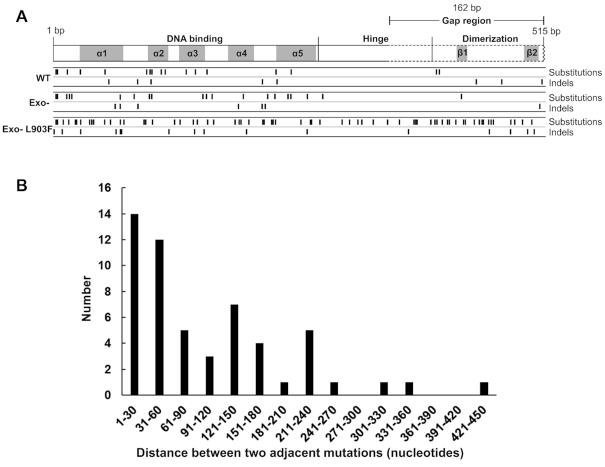
(**A**) Locations of base substitution and indels in the cI gene resulting in loss of repressor function for the WT, Exo- and Exo-L903F NtPOP^tom^ enzymes. Domains for DNA binding, hinge region and dimerization are shown. Alpha helices 1–5 (α1–5) and beta sheets (ß1–2) are indicated. (**B**) Distance between mutations in mutant cI genes replicated by the Exo-L903F NtPOP^tom^ enzyme.

We compared the recombinant NtPOP^tom^ enzymes to the well-studied Taq DNA polymerase, which lacks 3′-5 exonuclease activity (60,61). Following replication of the single-strand gap with the recombinant DNA polymerases, the replicated plasmids were transformed into *E. coli* cells and transformants selected on media supplemented with tetracycline or ampicillin. Samples of the replicated plasmids were treated with Hind III to monitor conversion of the single-stranded gap to newly replicated double stranded DNA (Supplementary Figure S3). The frequency of colonies containing plasmids with loss-of-function mutations in the *cI* gene was calculated by dividing the number of tetracycline resistant colonies by the number of ampicillin-resistant colonies (Table 1). The WT NtPOP^tom^ enzyme gave rise to the lowest frequency of mutant tetracycline colonies, which was about five-fold lower than those obtained with the Exo- enzyme and Taq DNA polymerase. The Exo-L903F enzyme gave rise to the highest frequency of tetracycline resistant colonies, which was 63-fold higher than that obtained with the WT NtPOP^tom^ enzyme. All plasmids sequenced from tetracycline-resistant colonies contained mutations in the *cI* gene verifying the absence of false positive colonies. The locations of the mutations in the *cI* gene (Figure 5A) showed that the recombinant NtPOP^tom^ enzymes were efficient strand displacement enzymes capable of displacing hundreds of base-paired nucleotides ahead of the replication fork. Mutations included changes to the ATG start codon located in a double-stranded region 353 base pairs beyond the single-stranded gapped region.

**Table 1.** Mutant frequencies and DNA polymerase error rates. Error rates in columns 5A and 5B were calculated from the data in columns 3 and 4 and Taq DNA error rates shown in brackets from: ^1^the supplier (New England Biolabs) and ^2^McInerney *et al.* (60). ^3^Column 5C error rates were from scoring mutations in the alpha 1 and 5 coding regions in the cI gene (this work). Columns 6D and 6E show relative error rates based on columns 5A and C respectively. nd- not determined

1		2	3	4	5			6	
					Error rates (mutations/base)			Relative error rates	
DNA polymerase		Mutant colony frequency	Relative mutant frequency	Average mutation no. per gene	A	B	C^3^	D	E
	WT	8.43 × 10^−5^	1.0	1.1	5.6 × 10^−5^	8.5 × 10^−6^	5 × 10^−6^	1	1
**NtPOP^tom^**	Exo-	4.50 × 10^−4^	5.3	1.1	3.0 × 10^−4^	4.5 × 10^−5^	4 × 10^−5^	5	8
	Exo-L903F	5.30 × 10^−3^	63	2.4	7.7 × 10^−3^	1.2 × 10^−3^	7 × 10^−4^	140	140
**Taq**		4.70 × 10^−4^	5.6	1.0	(2.85 × 10^−4^)^1^	(4.3 × 10^−5^)^2^	nd	5	nd

Mutant *cI* genes resulting from replication errors by the WT and Exo- NtPOP^tom^ enzymes contained an average of 1.1 mutations. This was raised to an average of 2.4 mutations in *cI* genes replicated by the Exo-L903F enzyme. Over 90% of mutant *cI* genes replicated using the WT and Exo- enzymes contained a single mutation (Supplementary Figure S9A). These were more common in the region encoding the N-terminal DNA binding region indicating the influence of sequence context on error frequencies and the location of codons essential for repressor function (Figure 5A). Replication by the Exo-L903F NtPOP^tom^ enzyme gave rise to multiple single base substitutions and/or single base indels at two to seven sites in about 50% of the *cI* genes sequenced (Supplementary Figure S9A-B). Exo-L903F mutations were distributed throughout the region replicated (Figure 5A). In *cI* genes with multiple mutations, 48% of mutations were located within 60 bases of each other and the frequency decreased with distance (Figure 5B). Ninety percent of these mutations were separated by over ten nucleotides and as much as 445 nucleotides. These multiple mutations in a single *cI* gene cannot be explained by replication errors from single events. The uneven pattern of decrease in frequency of adjacent mutations with distance observed is likely to reflect the influence of specific DNA sequences on the Exo-L903F error rate. Uneven distribution of replication errors due to sequence context is well established (62).

### Estimation of recombinant DNA polymerase error rates

Estimates of recombinant NtPOP^tom^ error rates were based on comparisons with Taq DNA polymerase. The Taq DNA polymerase error rate in the pH 8.8 buffer provided by the supplier (New England Biolabs) was 2.85 × 10^−4^ mutations per base, which is consistent with other reports (61,63). Variation in buffer composition and methods to measure error rates including different DNA replication templates have led to lower estimates, for example 4.3 × 10^−5^ (60). We used a pH 8.0 buffer, which was reported to reduce the Taq DNA polymerase error rate by around three-fold from 2.0 × 10^−4^ at pH 8.8 to 7.2 × 10^−5^ at pH 8.0 (63). Using Taq DNA polymerase high and low error rates as comparators provided an estimated error rate for the WT POP^tom^ enzyme that lies within a 7-fold range between 5.6 × 10^−5^ and 8.5 × 10^−6^ mutations per base (Table 1, columns 5A and 5B). Error rate determinations require identifying all the detectable sites within a sequence whose mutation would result in a defective protein (47). To provide an estimate of mutation rate based on the frequency of mutations in the *c1* gene we identified the detectable sites present in the region coding for 33 amino acids that include the alpha 1 and 5 helices (Supplementary Figure S4). Systematic studies on this region have identified the impact of changes to all 33 amino acids on repressor function (37,39). Analysis of *cI* mutations in the alpha 1 and 5 coding regions provided an estimated error rate for the WT NtPOP^tom^ enzyme of 5 × 10^−6^ mutations per base (Table 1 column 5C). Higher error rates of 4 × 10^−5^ and 7 × 10^−4^ were estimated for the Exo- and Exo-L903F enzymes, respectively (Table 1, column 5C). Error rate values for the recombinant NtPOP^tom^ enzymes based on mutations at detectable sites were in closer agreement with relative values calculated using the lower error rate of 4.3 × 10^−5^ reported for Taq DNA polymerase (Table 1, column 5B) (47,60). The relative error rates for the NtPOP^tom^ enzymes based on Taq DNA polymerase (Table 1, column 6D) and detectable sites in the *cI* gene (Table 1, column 6E) were in close agreement. The error rate was increased by five to eight-fold in the exonuclease deficient enzyme and by 140 fold in the Exo-L903F enzyme relative to the WT enzyme.

### Mutation spectrum of recombinant NtPOP^tom^ enzymes

**Figure 6.**
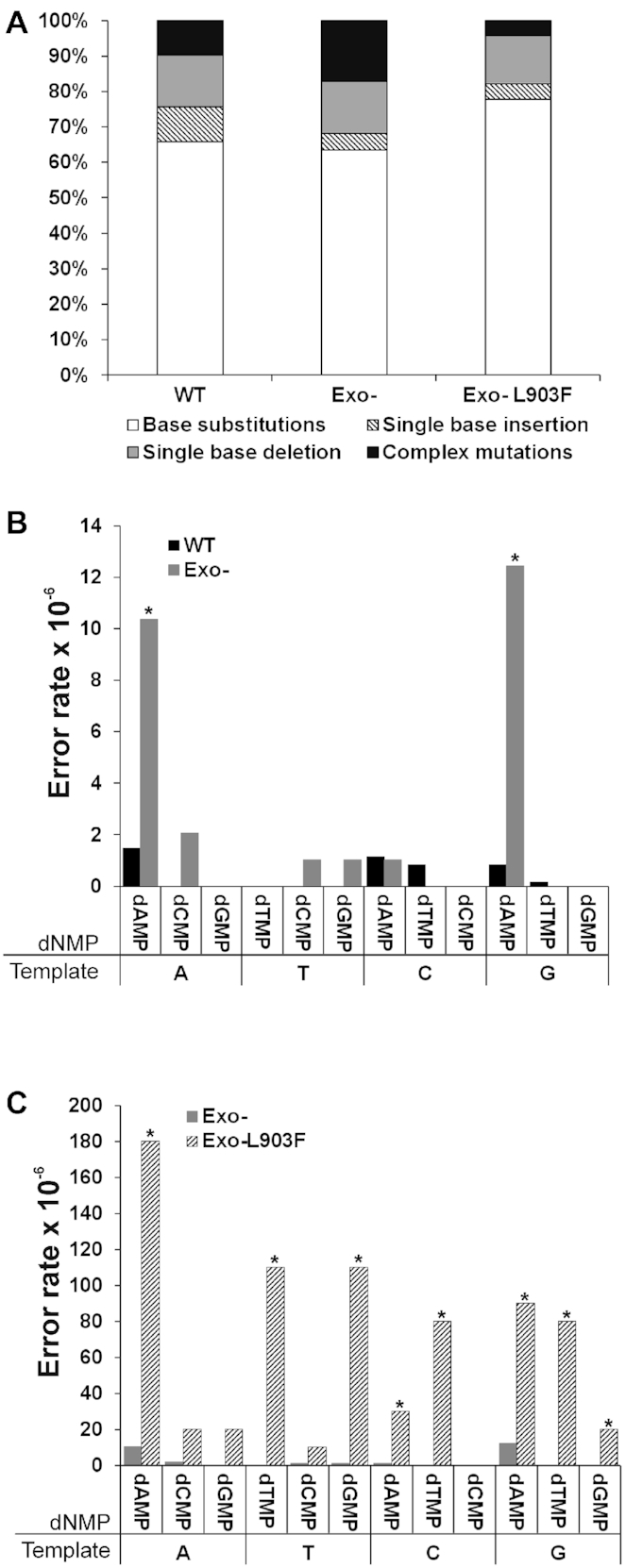
(**A**) Percentages of the different types of mutations associated with the WT, Exo- and Exo-L903F NtPOP^tom^ enzymes. No significant differences were found between the enzymes. Mispairing rates resulting in observed mutations for (**B**) WT and Exo- enzymes, (**C**) Exo- and Exo-L903F enzymes. Template base and mis-paired dNMP are indicated. Asterisks represent significant differences determined by chi squared tests (*P* < 0.001).

Base substitutions were the most common type of mutation and represented 66%, 63% and 78% of the *cI* mutations associated with WT, Exo- and Exo-L903F NtPOP^tom^ enzymes, respectively (Figure 6A, Supplementary Table S1). The percentage of transversion mutations were 70%, 85% and 68% for the WT, Exo- and Exo-L903F enzymes, respectively (Supplementary Figure S10). A common mismatch (A:A) shared by the WT and error-prone NtPOP^tom^ polymerases involved a template adenine mis-pairing with an incoming dATP (Figure 6B-C, supplementary Table S2). The Exo- enzyme also gave rise to a high proportion of G:A mispairings (Figure 6B). Other frequent mutations associated with the Exo-L903F enzyme arose from T:T, T:G, C:T, G:A and G:T mispairings (Figure 6C). Single base deletions were markedly more frequent than single base insertions for the Exo- and Exo-L903F enzymes (Figure 6A). Here, we define complex mutations as deletions/insertions of more than one base or substitutions of two adjacent bases, multiple base substitutions at closely spaced sites and a mixture of these changes (Supplementary Table S3). Complex mutations accounted for 10%, 17% and 4% of the total number of mutations for the WT, Exo- and Exo-L903F enzymes, respectively (Supplementary Table S1). For the WT enzyme, a complex mutation can be explained by deletion of a 6-base direct repeat by a slippage event. The majority of different types of mutations (base substitutions, INDELs and complex mutations) did not co-localise to the same position on the *cI* gene.

### Influence of WT enzyme on Exo-L903F mutation rate

**Figure 7.**
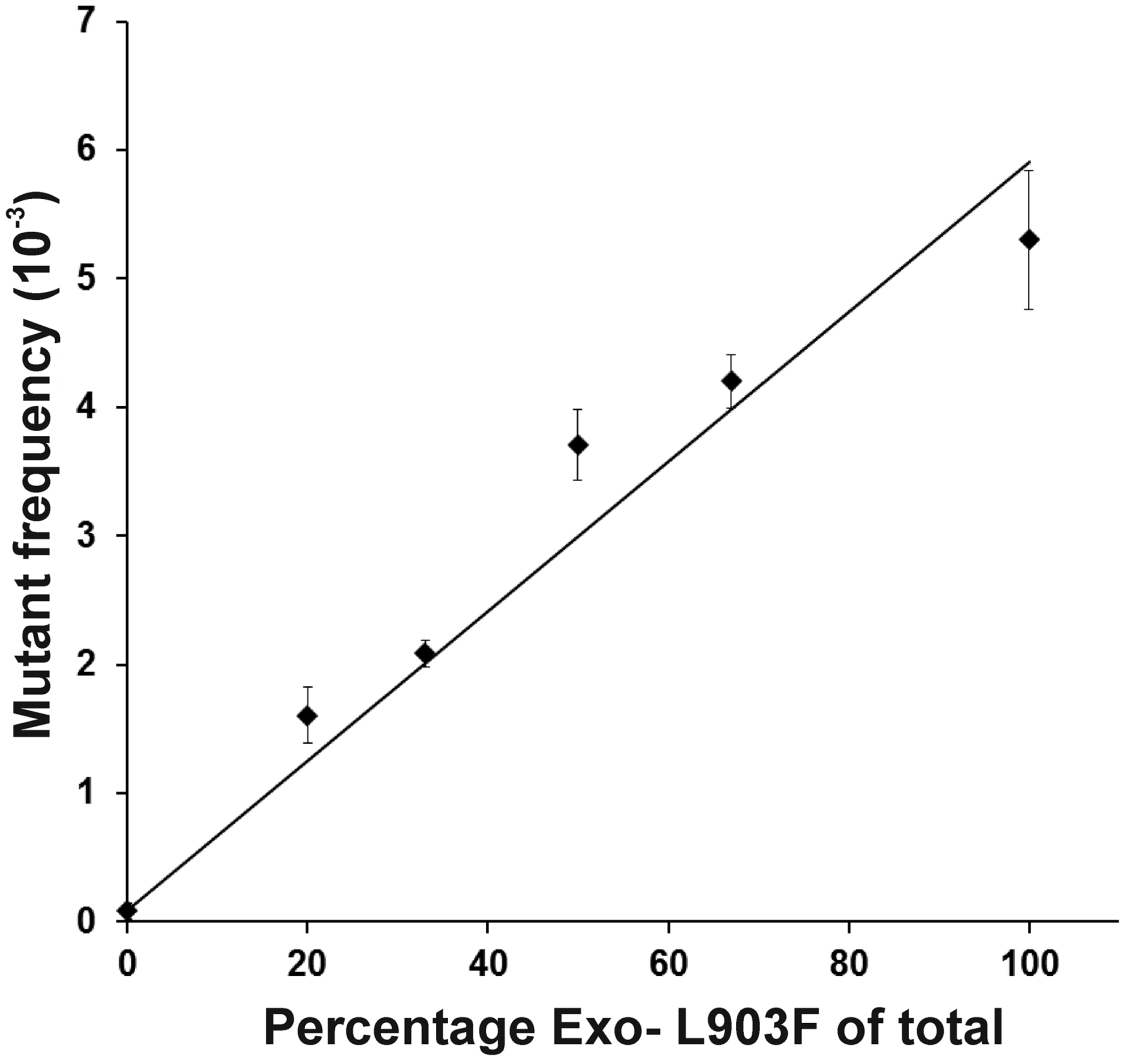
Relationship between mutant frequency and percentage of Exo-L903F enzyme in mixtures of Exo-L903F and WT NtPOP^tom^ enzymes. Exo-L903F and WT enzymes were combined in the proportions indicated to 3 pmol in total. Following replication of 30 fmol of gapped pUN121 with enzyme mixtures and transformation into bacteria, the mutant frequency was determined from the ratio of tetracycline to ampicillin resistant colonies. Results were from three independent replicates.

To evaluate potential interactions between the WT enzyme and the error-prone Exo-L903F DNA polymerase that might influence mutation rate, we tested mixtures of the two enzymes in the gap-filling replication assay (Figure 7). The results showed that mutant frequency increased in proportion to the amount of error-prone Exo-L903F present. The mutation rate was elevated even when the WT enzyme was in 4-fold excess. The data suggests that the error-prone enzyme is semi-dominant to the WT enzyme.

## DISCUSSION

Introducing amino acid substitutions into the exonuclease and polymerisation (L903F) domains of a tobacco POP produced a functional and highly error-prone enzyme. The WT NtPOP^tom^ enzyme had an estimated error rate of between 6 × 10^−5^ and 5 × 10^−6^ mutations per base. This was raised by 140-fold in the Exo-L903F enzyme. Removal of exonuclease activity alone increased the error-rate by 5–8-fold. *In vitro* competition experiments indicated the Exo-L903F enzyme was semi-dominant to the WT enzyme. High error rate and effective mutator activity in the presence of the WT enzyme makes the Exo-L903F enzyme a strong candidate for developing an organelle mutator system in plants. Mutation frequency was determined using a new genetic screen involving positive selection in *E. coli*, based on gain of tetracycline resistance (35).

Positive selection has the advantage of ease of isolation of resistant mutant colonies due to the absence of surrounding non-mutant bacterial colonies associated with mutant screens involving colour identification such as those based on the *lacZ* (41,42) or *cro* (40) genes. It also overcomes potential technical issues linked to poor development of colour resulting from uneven distribution of substrates such as 5-bromo-4-chloro-3-indolyl-β-d-galactopyranoside (X-gal) on solid media plates. Furthermore, the development of new genetic screens increases the number of template DNA sequences available for testing the fidelities of DNA polymerases. The assay involved *in vitro* replication of the coding sequence for the well-characterised bacteriophage lambda CI repressor protein (37,39). The assay showed the NtPOP^tom^ enzymes were efficient at displacing double stranded regions over 300 bp ahead of the replication fork. Previous work had shown that POPs were capable of displacing small 30 to 35 base oligonucleotides (18,19). Most single nucleotide mutations leading to loss of repressor function were found in the N-terminal DNA binding region of the repressor (37–39). This may reflect the influence of sequence context on POP error rates as well as the location of mutation sites resulting in loss of repressor function. Error rate estimates were determined from detectable sites in 99 nucleotides encoding the alpha 1 and 5 helices of the DNA binding domain. The estimated error rates for the recombinant NtPOP^tom^ enzymes based on mutations in the coding regions for alpha 1 and 5 helices were in reasonable agreement with the values calculated using relative mutation frequency and error rate for Taq DNA polymerase. Closer agreement was found with calculations based on the lower range of estimated error rates reported for Taq DNA polymerase, which vary from ∼3 × 10^−4^ to 4 × 10^−5^ (60,61). Here, we used a pH 8.0 buffer which has been shown to reduce Taq DNA polymerase error rate compared to the standard conditions of pH 8.8 (63). Error rates vary from 10^−3^ for low fidelity enzymes to 10^−6^ for high fidelity enzymes (62). The WT NtPOP^tom^ with an error rate of 6 × 10^−5^ to 5 × 10^−6^ would appear to be a medium to high fidelity enzyme similar to the Klenow fragment of *E. coli* Pol I with an error rate of 6 × 10^−6^ (55). The error rate of the WT NtPOP^tom^ enzyme was not too dissimilar from the error rate of 7.3 × 10^−5^ reported for the *A. thaliana* POP AtPolA, which is proposed to be the main replicative enzyme in *A. thaliana* organelles (27). The AtPolB paralog with a higher reported error rate of 5.45 × 10^−4^ is considered to have a predominant role in repair (27).

Loss of 3′–5′ exonuclease activity increased the error rate of the NtPOP^tom^ Exo- enzyme by 5–8-fold which was comparable to the 4 to 7 fold increase in error rates reported for 3′–5′ exonuclease-deficient derivatives of the Klenow fragment (53,55). This was higher than the 1.3- to 1.7-fold increase in error rates reported for the 3′–5′ exonuclease deficient *A. thaliana* organellar DNA polymerases using *lacZ* as the template (27). The data may indicate variation in the importance of the exonuclease domain of POPs in different plant taxa. The limited impact of removing exonuclease activity on POP error rates contrasts with the much larger error rate increases observed for exonuclease deficient gamma DNA polymerases used as mitochondrial mutators (3,6,64). This reflects a fundamental difference between the DNA polymerases present in animal and fungal mitochondria versus those present in the organelles of other taxa. A 20-fold increase in error rate was reported for the 3′–5′ exonuclease-deficient human mitochondrial gamma DNA polymerase (64). To reduce the fidelity of the NtPOP^tom^ enzyme beyond the 5- to 8-fold decrease achieved by ablating exonuclease activity we introduced the L903F substitution into the polymerisation domain. Discrimination of the correct nucleotide during polymerisation is the major determinant of replication fidelity (65). Combining a defective exonuclease domain with a L903F substitution in the polymerisation domain of the NtPOP^tom^ enzyme raised the mutant frequency by 63-fold and error rate by about 140 fold. By comparison, combining mutations in the exonuclease and polymerisation domains of *E. coli* Pol I raised the mutation rate by around 400-fold (53). The highly error prone NtPOP^tom^ Exo-L903F enzyme exhibited reduced DNA synthesis activity compared to the WT and Exo- enzymes. This is in contrast to the results obtained with the Klenow fragment of *E. coli* DNA Pol I in which the equivalent I709F substitution did not impact on DNA synthesis activity (53) but is consistent with a reduction in DNA synthesis reported for the equivalent L979F substitution in Pol ζ, which is a family B polymerase (49). The native NtPOP^tom^ enzyme contains a C-terminal lysine residue (Supplementary Figure S6). All recombinant NtPOP^tom^ enzymes contained this C-terminal lysine followed by a linker peptide (GSGSGS) and C-terminal Strep-tag II (WSHPQFEK). The potential influence of the tag on activity was not investigated. In the distantly related bacteriophage T7 DNA polymerase, replacement of the C-terminal histidine with alanine reduces the activity of the enzyme (66).

About half of the mutant *cI* genes replicated by the Exo-L903F enzyme contained a single mutation whereas the remainder contained multiple mutations varying from two to seven (Supplementary Figure S9A, B). The frequency of *cI* genes with multiple mutations was much higher than the product of the frequency of *cI* genes with single mutations. This rules out their origin from independent events and can be explained by replication models in which the first error increases the probability of a DNA polymerase introducing further replication errors (49). Multiple mutations per replicated template is a feature associated with low fidelity enzymes such as translesion DNA polymerases (40,49). POPs have been identified as translesion DNA polymerases (19) and the Exo-L903F enzyme is a highly error prone POP derivative. Whilst the frequency of two adjacent mutations made by the Exo-L903F enzyme reduced with the length of the intervening sequence, 35% of the mutations were separated by over 100 nucleotides. The propensity of Exo-L903F to make multiple mutations *in vitro* may be a useful characteristic to monitor the action of mutagenesis by the enzyme in plant organelles.

Sequencing mutant *cI* genes showed that seventy-eight percent of the mutations associated with the NtPOP^tom^ Exo-L903F enzyme were base substitutions of which 68% were transversion mutations. Frequent A:A mispairings of template to dNMP were common to WT and error prone NtPOP^tom^ enzymes (Supplementary Table S2). This gave rise to T → A transversions in the synthesized strand. For the Exo-L903F enzyme, A:A and T:T mispairings accounted for 58% of the total transversion mutations. T:T mispairings were also a feature of a mutant *E. coli* DNA Pol I lacking exonuclease activity and containing a I709F substitution in the polymerisation domain (53). Exo-L903F gave rise to single base deletions at a 3-fold higher frequency than single base insertions, which was similar to the properties of many other DNA polymerases (53,62). In the assay, Taq DNA polymerase showed a preference for A to G substitutions resulting from a template thymine mispairing with a guanine in the *cI* gene (Supplementary Table S2). This was consistent with previous results showing that base substitutions involving T:G mispairings are the most frequent for Taq DNA polymerase (60,61).

Genetic screens using *E. coli* to identify errors introduced during *in vitro* replication of DNA templates by DNA polymerases have provided a robust method to assay the fidelities and mutation spectra of DNA polymerases. The results from these genetic screens obtained over several decades support errors introduced during *in vitro* replication by DNA polymerases as the primary causes of the mutation patterns observed (40,42,47,54,55,67). We used a *recA* mutant in common with other studies (40,42,47). Complex mutations involving more than one nucleotide have been previously documented using genetic screens (40,49,55). These mutations were associated with the NtPOP^tom^ enzymes but not Taq DNA polymerase. As far as we are aware the potential contribution of bacterial repair pathways to complex mutations, which was not the main focus of this work, has not been investigated in previous studies. The use of alternative *E. coli* strains such as the low mutation rate MDS42pdu strain (68) could be used to study this theoretical possibility. The influence of plant organelle DNA repair pathways on the mutation spectrum of the NtPOP^tom^ Exo-L903F enzyme requires the transformation of this enzyme into plants. Comparison of the mutation spectra from the *in vitro* data obtained from replication of the *cI* gene (this work) with *in vivo* data obtained by expressing the NtPOP^tom^ Exo-L903F enzyme in plant organelles, will improve our understanding of organelle genome maintenance pathways in plants.

## Supplementary Material

gkaa929_Supplemental_File
