# Supplementary material for: Construction of a highly error-prone DNA polymerase for developing organelle mutation systems
Source: Nucleic Acids Res. 2020 Nov 2;48(21):11868–79. doi: 10.1093/nar/gkaa929 (PMC7708058; doi:10.1093/nar/gkaa929)
Supplement: gkaa929_Supplemental_File [file gkaa929_supplemental_file.pdf]

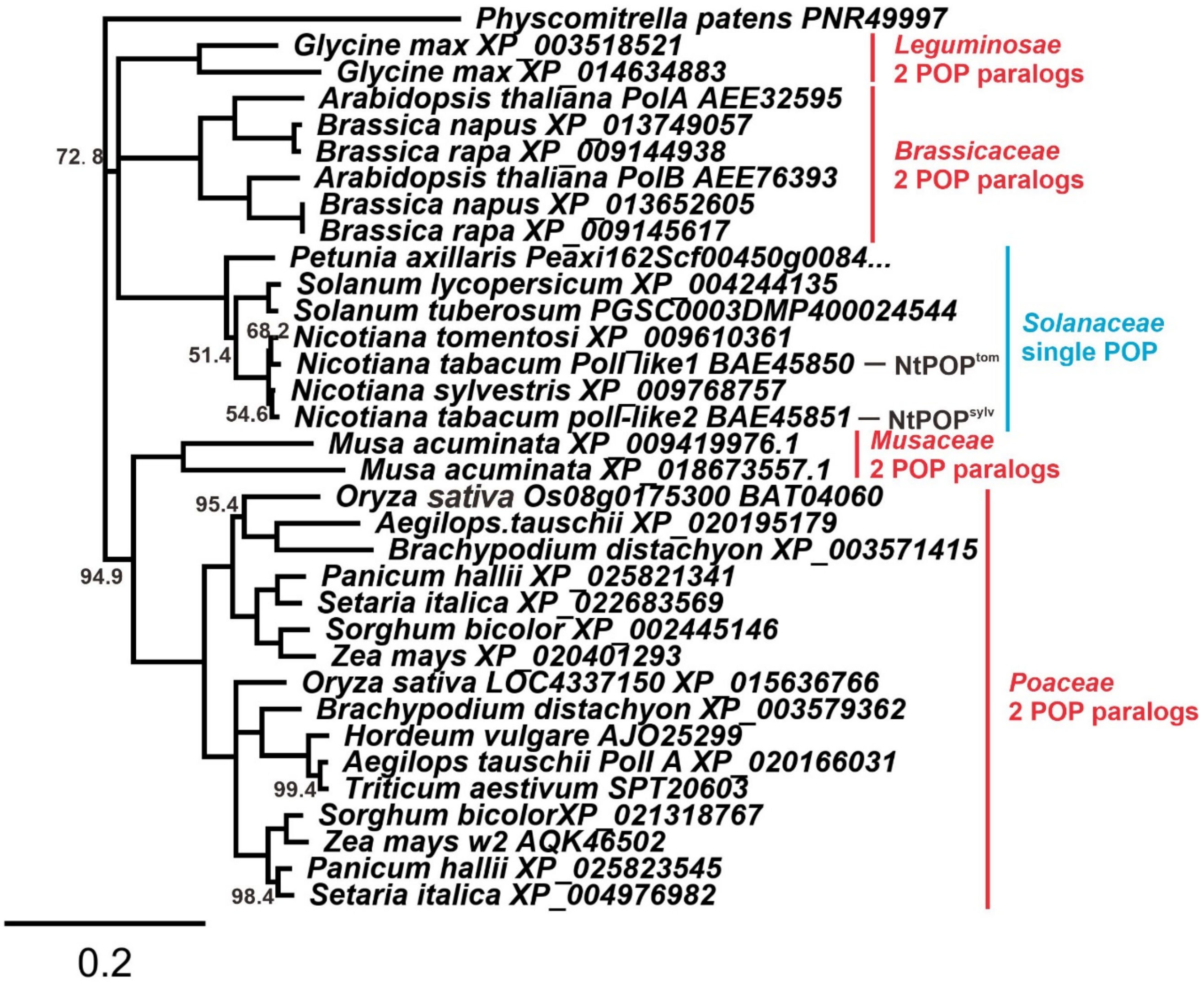

Supplementary Fig. S1. Neighbour joining consensus tree of indicated POP sequences. Bootstrap values (1000 replicates) were 100% unless indicated at nodes. *Physcomitrella patens* was the outgroup. Sequences with indicated accession numbers were retrieved from GenBank. *Brassicaceae, Muscaceae, Poaceae, Leguminosae*, *Solanaceae* families are indicated. All families shown contain two POP paralogs apart from the *Solanaceae*, which contains a single POP. Scale bar: amino acid substitutions per site.

Supplementary Fig. S2. Recombinant NtPOP ^{tom} DNA sequence. Domains and base substitutions for Exo- and L903F changes are shown.

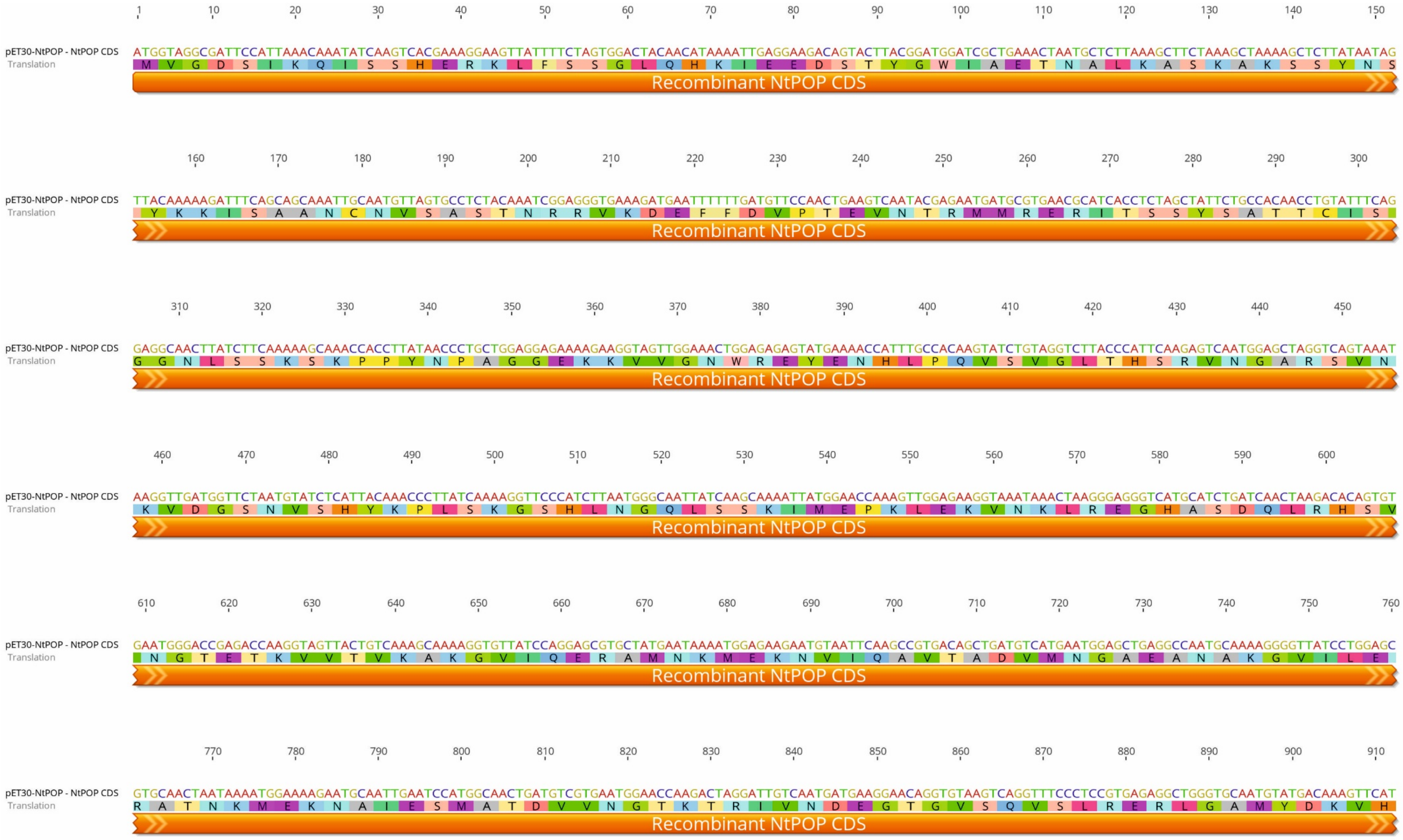

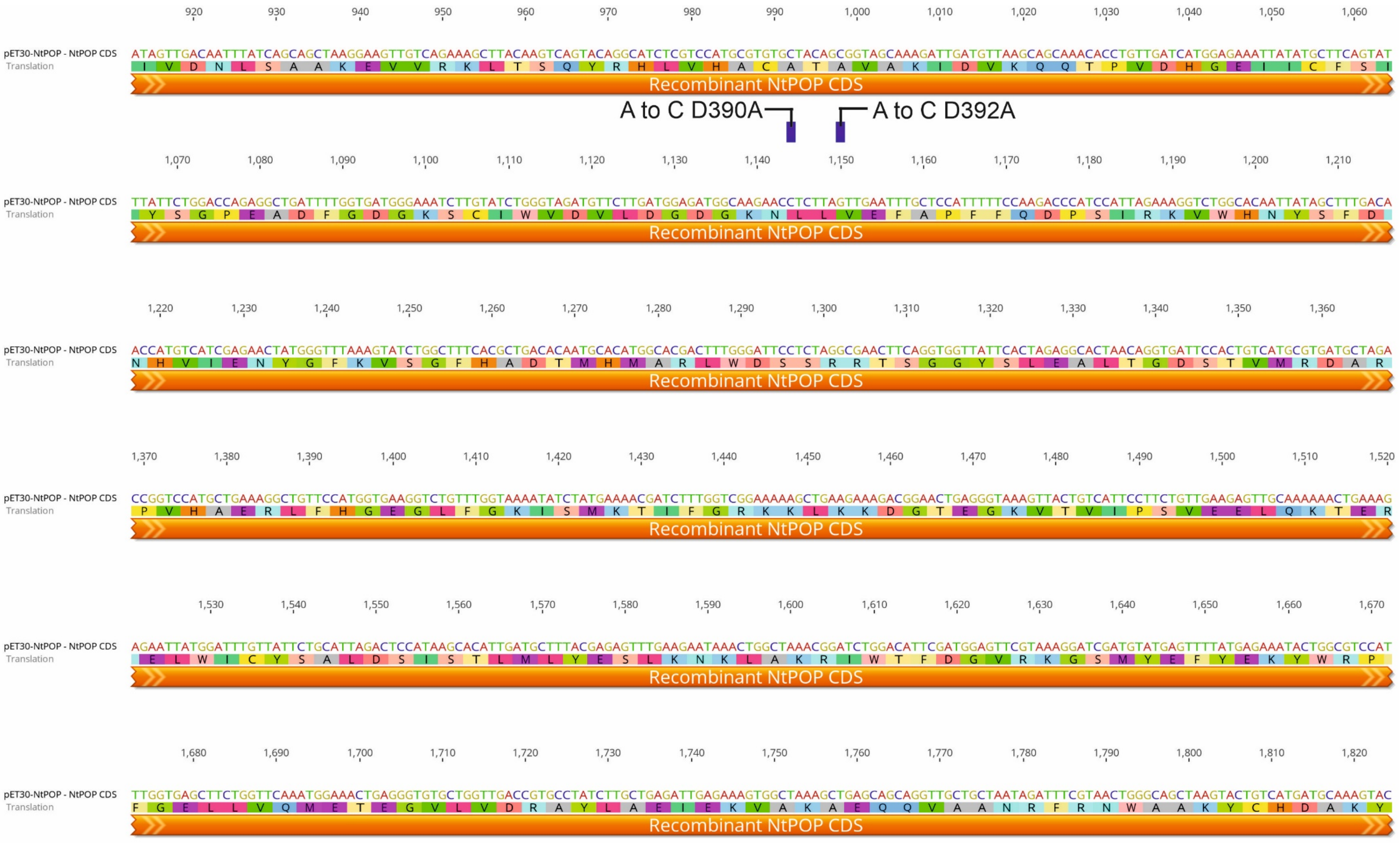
pET30-NtPOP - NtPOP CDS
Translation
Recombinant NtPOP CDS
A to C D390A
A to C D392A

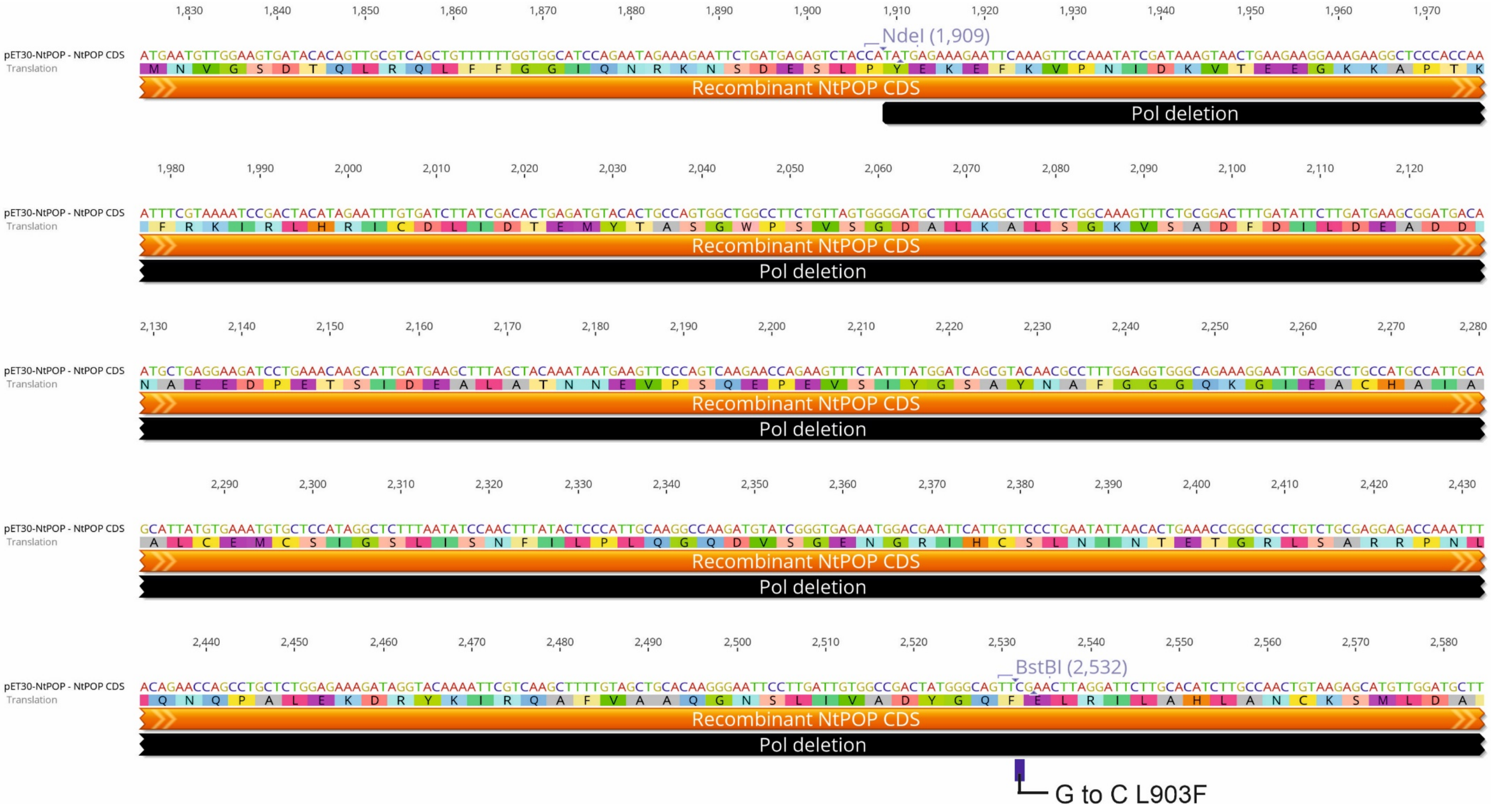
pET30-NtPOP - NtPOP CDS
Translation
NdeI (1,909)
Recombinant NtPOP CDS
Pol deletion
BstBI (2,532)
G to C L903F

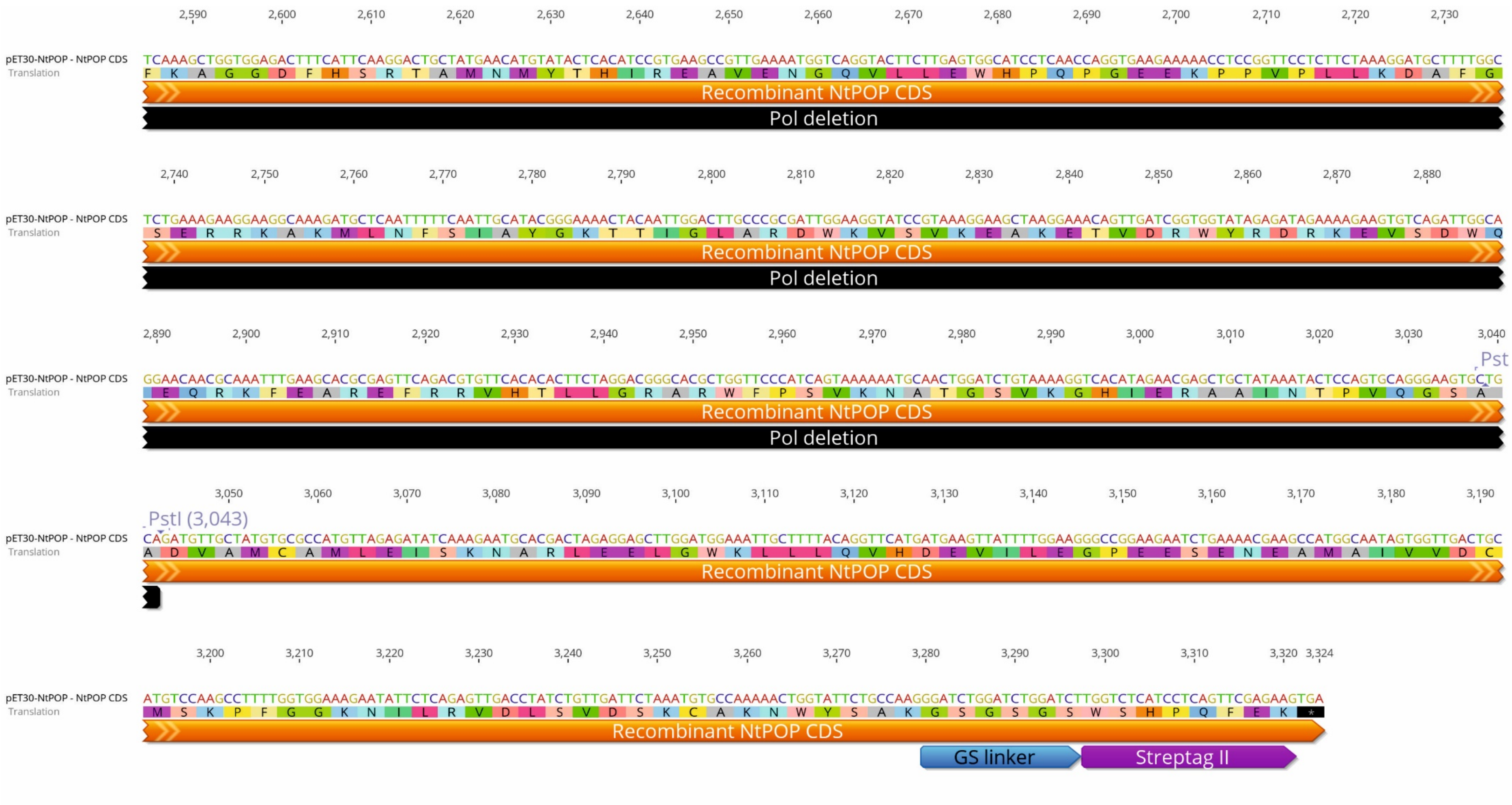
pET30-NtPOP - NtPOP CDS
Translation
Recombinant NtPOP CDS
Pol deletion
PstI (3,043)
GS linker
Streptag II

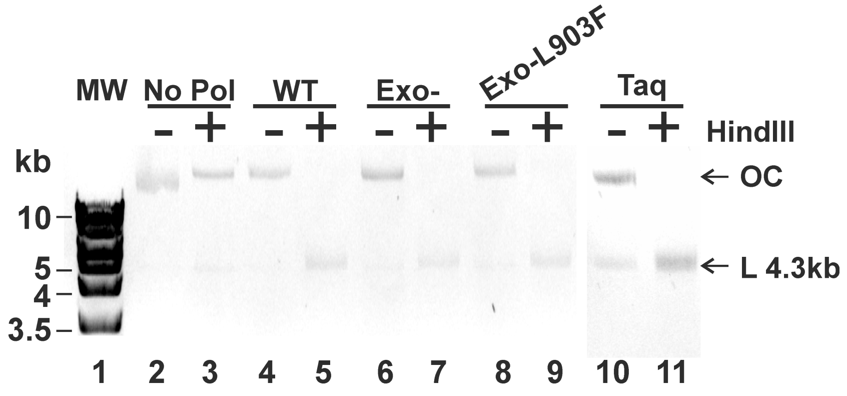

Supplementary Fig. S3. Agarose gel showing gapped pUN121 after the replication step with the indicated DNA polymerases before (-) and after (+) treatment with Hind III. MW standards, linear (L) and open circular (OC) pUN121 bands are indicated.

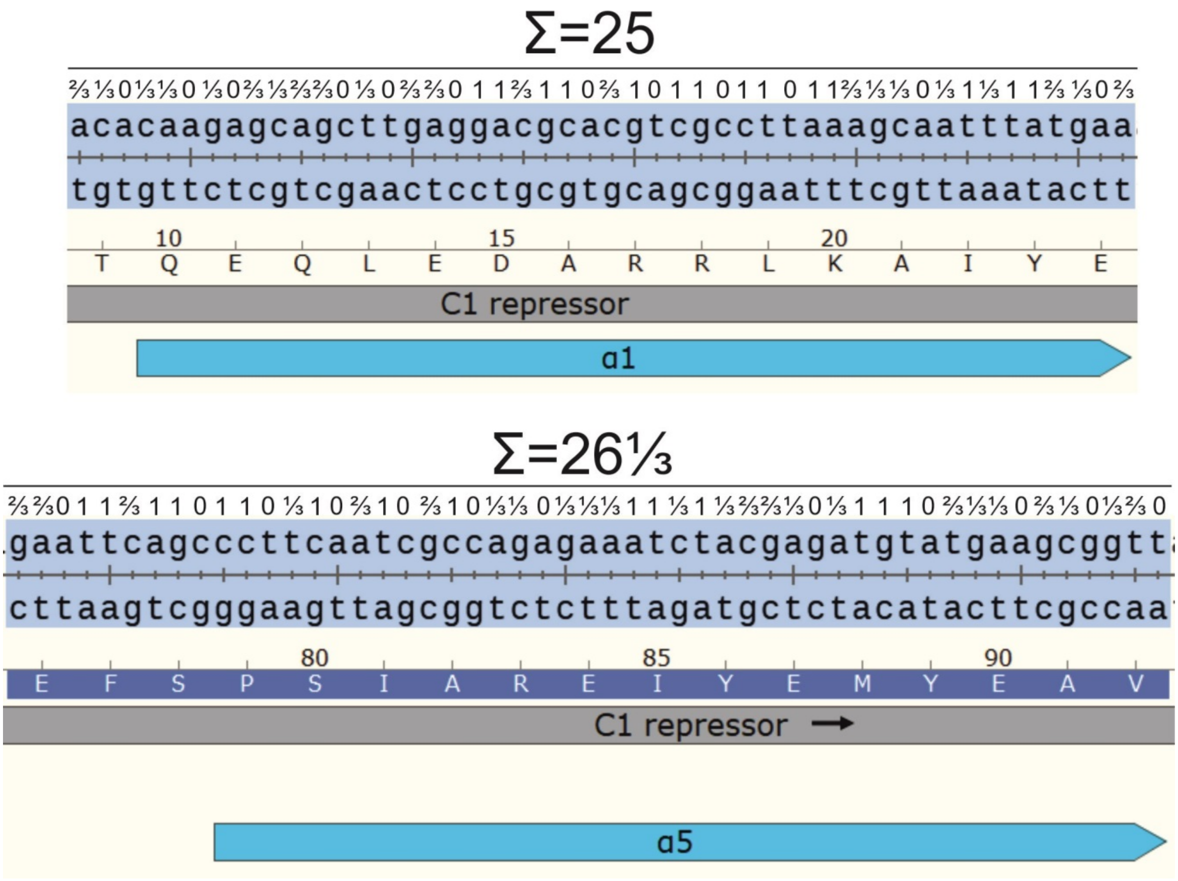

Supplementary Fig. S4. Region of the phage lambda *cI* gene encoding alpha helices 1 and 5. Error rate calculations require an estimate of detectable sites at which a base substitution gives rise to a detectable phenotype (1,2), which in this case is loss-of-repressor function giving rise to a tetracycline resistant phenotype. At each position the fraction of base substitutions giving rise to a loss of repressor function is indicated above the base shown. Amino acids amenable and not amenable to changes that retain repressor function have been documented in detail (3,4). Positions at which all three potential base substitutions do not affect repressor function are scored as zero, whilst positions at which all three base substitutions result in loss of function are scored as one. Positions at which only one base substitution or two base substitutions results in loss of function are scored as 1/3 and 2/3, respectively. The sum of all the base substitutions giving rise to loss-of-function is the number of detectable sites within this coding region of 99 nucleotides. Indel mutations resulting in frameshift mutations would be detected at all 99 nucleotides.

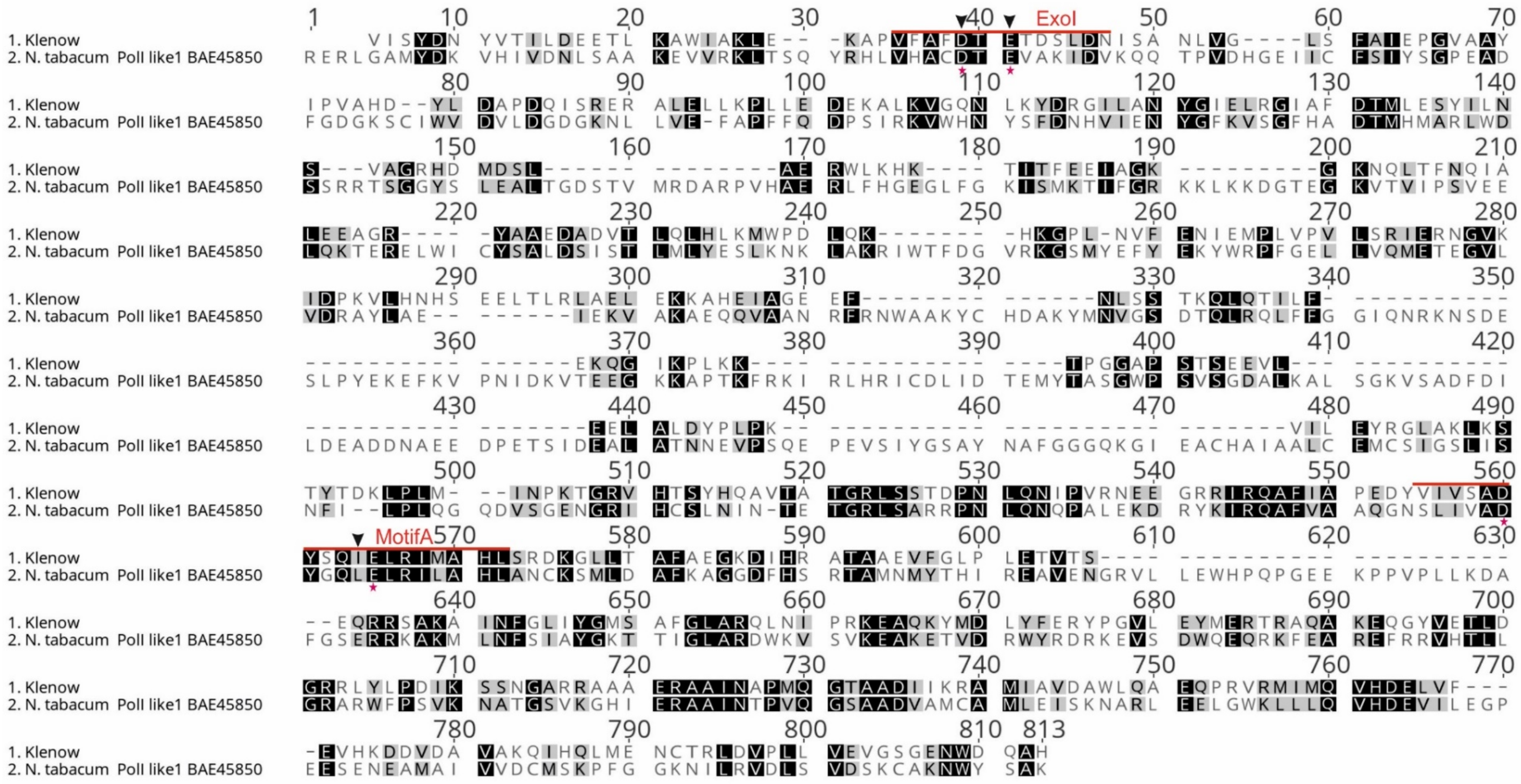

Supplementary Fig. S5. Alignment of the Klenow fragment of *E. coli* DNA Polymerase 1 with *N. tabacum* PolI-like1 (NtPOP^{tom}). Conserved amino acids in family A DNA polymerases are labelled with stars. The Motif Exo I and Motif A are indicated by red lines. Amino acid substitutions within these motifs are indicated by black arrow heads. The presequence and disordered region (1 – 352) were removed from NtPolI-like1 (NtPOP^{tom}). Identical amino acids are shaded black and similar amino acids are shaded grey.

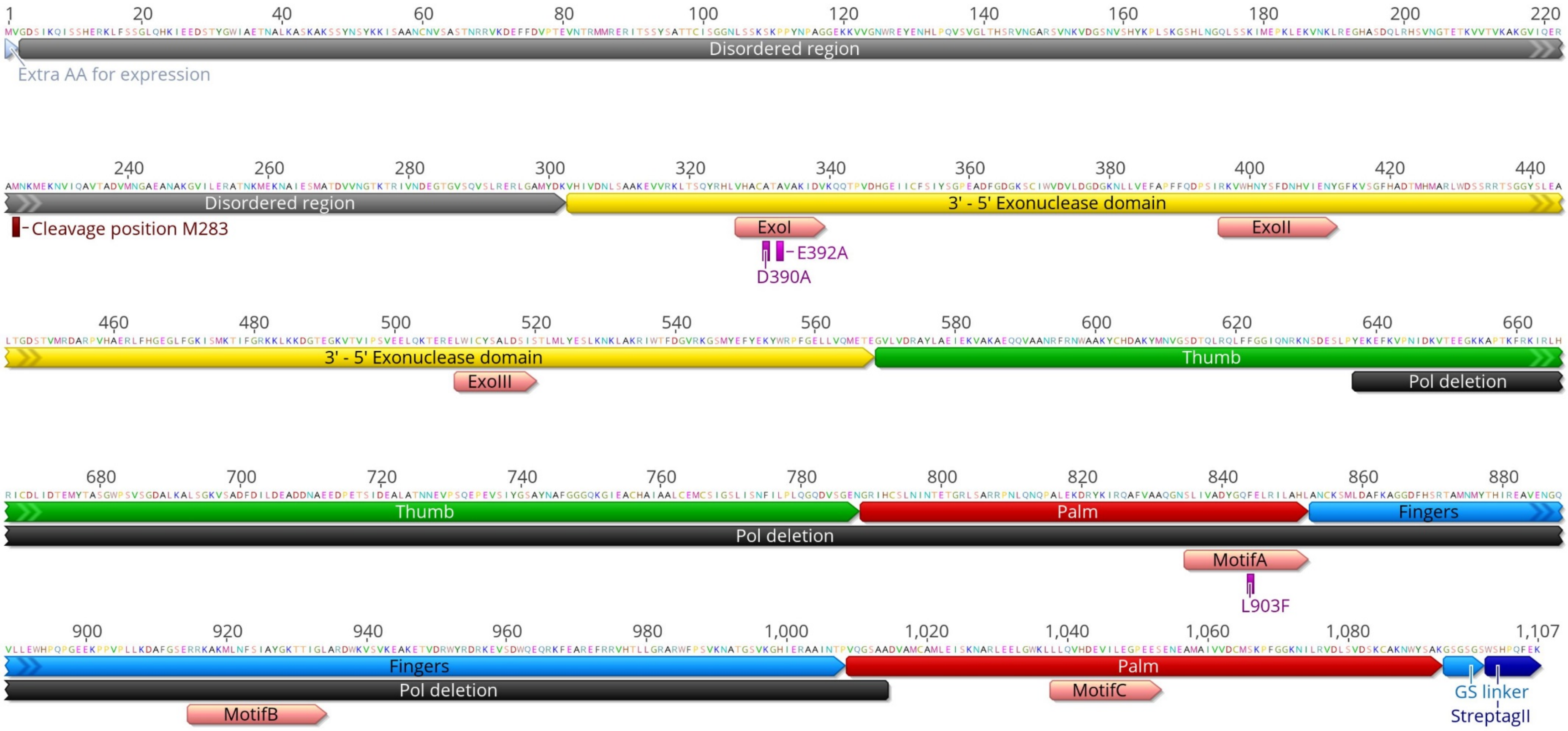

Supplementary Fig. S6. Recombinant protein sequences showing disordered sequence, 3’-5’ exonuclease and 5’-3’ polymerisation domains. The presequence was replaced with Met Val. Shown are: proteolytic cleavage site, amino acids 331 and 333 corresponding to the D390A and E391A substitutions in the full length protein; amino acid 844 corresponding to the L903F substitution; region deleted in Pol- ; C-terminal GS linker and Streptag II.

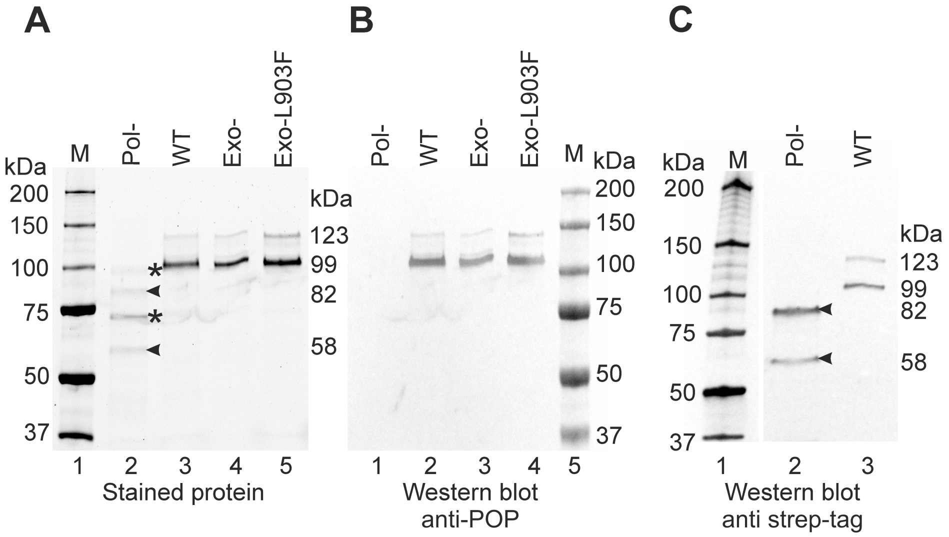

Figure S7. SDS-PAGE analyses of purified recombinant NtPOP^{tom} enzymes**. (A)** Total protein visualised with Bio-Rad (Watford, UK) tri-halo compound-based stain-free method. Protein blot analysis with **(B)** POP-specific polyclonal antibody, and **(C)** Strep tag specific monoclonal antibody. Shown are samples loaded, Pol- bands (arrow heads), bands lacking a strep-II tag (asterisks), MW size standards and sizes of bands in recombinant NtPOP^{tom} lanes.

Explanatory text: The purified WT, Exo- and Exo-L903F recombinant proteins fractionated by SDS-PAGE appeared as two bands (Fig S7A, lanes 3-5). Both bands bound to a POP-specific antibody raised against a peptide in the palm region (see location in Fig. 2A) of the polymerisation domain (Fig S7, lanes 2-4). The larger band corresponded to the intact 1107 amino acid recombinant protein (123 kDa). Cleavage within the disordered region gives rise to the smaller 870 amino acid (99 kDa) band with methionine 283 at the N-terminus as determined by N-terminal sequencing. This 99 kDa protein contains the 3'-5' exonuclease and polymerisation domains required for function (Fig 2A). The 737 amino acid (82 kDa) Pol- protein accumulated to much lower amounts than the longer recombinant proteins hindering its purification. Multiple bands were visualised in Pol- preparations by SDS-PAGE (Fig S7A, lane 2). These bands included the predicted 82 kDa Pol- protein and a 58 kDa protein resulting from cleavage within the disordered region, which contained the Strep II tag and bound to Strep-Tactin (arrowed in Fig S7A and S7C, lane 2). These bands lack the polymerisation domain and did not bind the antibody recognising this region (Fig S7B, lane 1). The other bands (Fig S7A, lane 2, bands marked*) did not bind a monoclonal antibody recognising Strep tag-II (Fig S7C, lane 2) and probably represent contaminating bacterial proteins. Pol- is an inactive recombinant protein and provided a negative control to verify the removal of bacterial DNA polymerases by our purification regime.

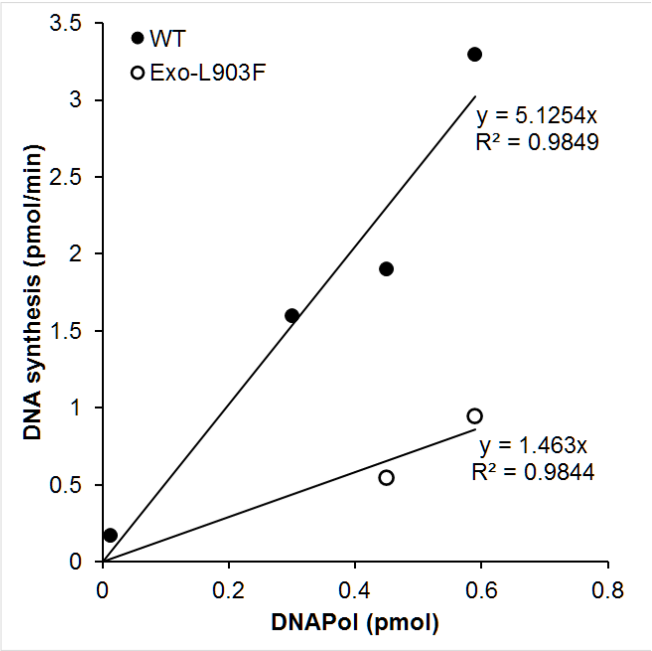

Supplementary Fig. S8. DNA Polymerase Specific Activity. Synthesis of double-stranded DNA was from a 35 base oligonucleotide (M13-F, Supplementary Table S4) annealed to single-stranded M13mp18 DNA. The activity of the Exo- L903F enzyme was approximately 30% of the wild type enzyme (WT).

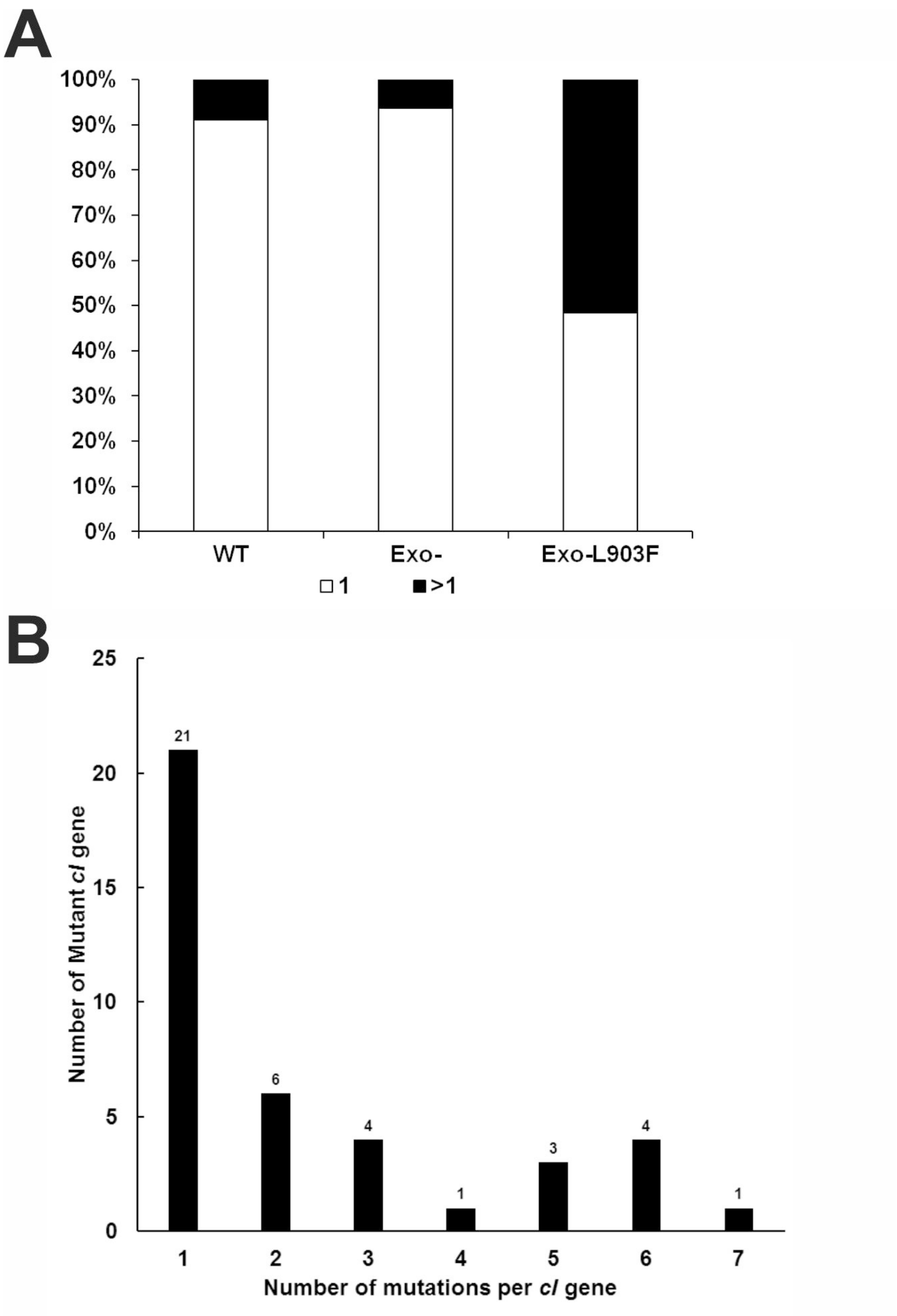

Supplementary Fig. S9. A. Percentage of mutant *cI* genes with single and multiple mutations for indicated NtPOP^{tom} enzymes. Significant differences were found for the Exo- L903F enzyme relative to the WT and Exo – enzymes ($p < 0.05$). The differences between WT and Exo– enzymes were not significant ($p < 0.05$) **B.** Number of mutant *cI* genes with the indicated number of mutations at separated sites after replication by the NtPOP^{tom} Exo- L903F enzyme.

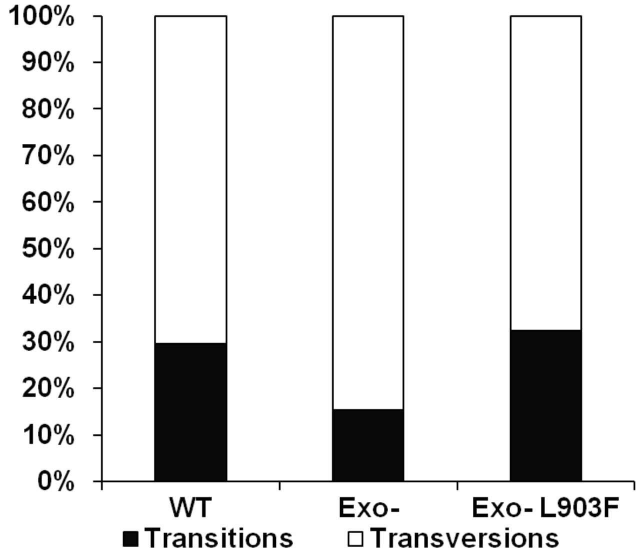

Supplementary Fig. S10. Percentage of transition versus transversion base substitutions for the indicated recombinant $NtPol^{tom}$ enzymes. Chi-squared tests showed no significant differences between the enzymes for $p < 0.05$.

References

1. Kunkel, T.A. and Alexander, P.S. (1986) The base substitution fidelity of eucaryotic DNA polymerases: Mispairing frequencies, site preferences, insertion preferences, and base substitution by dislocation. *J Biol Chem*, **261**, 160-166.
2. Keith, B.J., Jozwiakowski, S.K. and Connolly, B.A. (2013) A plasmid-based lacZα gene assay for DNA polymerase fidelity measurement. *Anal Biochem*, **433**, 153-161.
3. Reidhaarolson, J.F. and Sauer, R.T. (1988) Combinatorial cassette mutagenesis as a probe of the informational content of protein sequences. *Science*, **241**, 53-57.
4. Sauer, R.T. (2013) Mutagenic dissection of the sequence determinants of proteinfolding, recognition, and machine function. *Protein Sci*, **22**, 1675-1687.

Supplementary Table S1. Number (percentage) of types of mutations found in mutant *cI* genes replicated by the recombinant WT, Exo- and Exo- L903F NtPOP^{tom} enzymes.

Enzyme	WT	Exo-	Exo-Pol903F
**Sequenced mutants**	37	38	40
**Total number of mutations**	41	41	95
Base substitutions	27 (66%)	26 (63%)	74 (78%)
Single base insertions	4 (10%)	2 (5%)	4 (4%)
Single base deletions	6 (15%)	6 (15%)	13 (14%)
Complex mutations	4 (10%)	7 (17%)	4 (4%)

Supplementary Table S2. Number of different mutation types found in mutant c1 genes replicated by the recombinant WT, Exo- and Exo- L903F NtPOP^{tom} enzymes. Details of single base indels are shown in Table S1 and complex mutations in Table S3.

**DNA Polymerase** (No. *cI* genes sequenced)	**Synthesised strand mutation**	**Number found**	**Mismatch formed** (Template:dNMP)
**Taq DNA polymerase** (11)	A→G	7	T:G
	C→A	1	G:A
	C→T	1	G:T
	G→A	1	C:A
	G→T	1	C:T
**NtPOP** (37)	T→A	9	A:A
	C→A	5	G:A
	C→T	1	G:T
	G→A	7	C:A
	G→T	5	C:T
	Single indels	10	
	Complex mutations	4	
**Exo-** (38)	A→C	1	T:C
	A→G	1	T:G
	T→A	10	A:A
	T→C	2	A:C
	C→A	11	G:A
	G→A	1	C:A
	Single indels	8	
	Complex mutations	7	
**Exo- L903F** (40)	A→T	11	T:T
	A→G	11	T:G
	T→A	18	A:A
	T→C	2	A:C
	T→G	2	A:G
	C→A	9	G:A
	C→T	8	G:T
	C→G	2	G:G
	G→A	3	C:A
	G→T	8	C:T
	Single indels	17	
	Complex mutations	4	

Supplementary Table S3. Details of mutations classified as ‘complex’ in mutant *cI* genes replicated by the recombinant WT, Exo- and Exo-L903F NtPOP^{tom} recombinant enzymes. Changed bases in red. Double red lines indicate deleted bases. Underline indicates bases replaced with those shown above the sequence.

Enzyme	Number of Complex Mutations	Sequence of Synthesised Strand Showing Mutations
Taq DNA Polymerase	Not detected	
WT NtPOP^{tom}	4	1. GACTGCCCCATC~~CCCATC~~TTGTCTGCGA<br>2. TTCTTCAACG~~CTAAC~~TTTGAGAA<br>3. AAATTGCT~~TT~~AAGGCG<br>TTG<br>4. ACCAACGCCTGACTGCCCCATCCCCATCTTG
Exo- NtPOP^{tom}	7	A A A A<br>1. GCTTCATACATCTCGTAGATTTCTCT<br>A<br>2. GGCGAT~~TG~~AAGGGCT<br>3. CATTAA~~ATAAAGCACCAA~~CGCCTGA<br>4. ATCTTGT~~CT~~GCGAC<br>5. CTCTGG~~CG~~ATTGAA<br>6. CAGAATC~~AC~~TGGCTTT<br>AT<br>7. CATTTTTCTTTTTTTT
Exo- L903F NtPOP^{tom}	4	T T<br>1. TCTCTGGCGATTGAAG<br>G T<br>2. AACGCTAACTTTGA<br>3. CTGC~~T~~C~~A~~GGGT<br>AA<br>4. CTTTTTTGTGCT

Supplementary Table S4. Oligonucleotides used (Sigma-Aldrich, Southampton)

**Name**	**Sequence 5' → 3'**
delNdeIPstI-*F*	TACGAGAAAGAATTCAAAGTTGCA
delNdeIPstI-R	ACTTTGAATTCTTTCTCG
M13-F	TTCCCAGTCACGACGTTGTAAAACGACGGCCAGTG
pUN121-F	CCCCCAAGTCTGGCTATGCA
pUN121-R	CAACCATTATCACCGCCAGAG
CompetpUN-R1	GCTTAGAACCTTTACCAAAGGTGATGCGGAGAGATGGGTAAGCACAACCAAAAAAGC
CompetPUN-R2	CAGTGATTCTGCATTCTGGCTTGAGGTTGAAGGTAATTCCATGACCGCACCAA
CompetPUN-R3	CAGGCTCCAAGCCAAGCTTTCCTGACGGAATGTTAATTCTCGTTGACCCTGA
pUN121_mut	TCAGGGTCAACGAGAATTAACATTCCGTCAGGAGCTTGGCTTGGAGCCTG
